# Understanding the Influence of a Water Molecule in the Structure of a Dimer

**DOI:** 10.1021/acsomega.5c11915

**Published:** 2026-02-06

**Authors:** Fernando Torres-Hernández, Paúl Pinillos, Ander Camiruaga, Imanol Usabiaga, José A. Fernández

**Affiliations:** Department of Physical Chemistry, Faculty of Science and Technology, 73038 University of the Basque Country (UPV/EHU), Barrio Sarriena s/n, Leioa 48940, Spain

## Abstract

Understanding how water influences molecular aggregation is essential for interpreting noncovalent interactions in biological and chemical systems. In this study, we investigate the solvation of organic dimers formed by 2-phenylethanol (PEAL), 2-phenethylamine (PEA), and 2-phenethylthiol (PET) through a combination of two-color REMPI and IR-UV double resonance spectroscopy, supported by quantum chemical calculations. Despite the structural and electronic differences among the three molecules, all systems—except PET_2_—form similar monohydrated structures, where water adds to the preexisting hydrogen bond network rather than inserts into it. The observed behavior seems to follow qualitative energetic trends, with the most stable structures favored under the experimental conditions; structural accessibility could also influence the formation of certain isomers. Spectroscopic shifts in OH, NH, and SH stretching regions reveal correlations between OH···S and OH···N interactions, despite their differing electronic characteristics, while the OH···O interaction needs to be stronger to induce comparable shifts. Computational analysis confirms that the experimentally observed structures correspond to the most stable isomers, although alternative cyclic structures were also identified with slightly higher energies. By systematically comparing dimers formed by OH-, NH-, and SH-containing molecules, this work extends previous studies on water insertion versus addition and provides new insights into how donor type influences hydration patterns and spectroscopic signatures.

## Graphical abstract

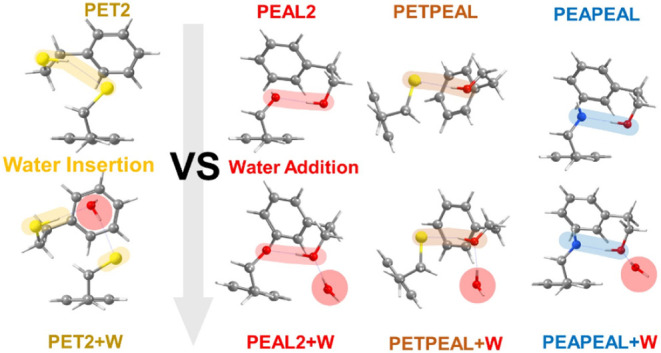

Molecular aggregation is driven by the ability of molecules to establish noncovalent interactions. Although these forces are challenging to model and predict due to their wide range and small relative value, the extensive experimental data obtained, primarily by molecular spectroscopy in supersonic expansions, have allowed computational scientists to refine the quantum mechanics-based algorithms and to turn them into highly accurate predictive tools. Still, the inclusion of water in the system results in a substantial increase in complexity. While the final structure (or structures) that a dimer may adopt typically depends on the formation of hydrogen bonds, followed by other secondary interactions, such as π···π or the interaction between aliphatic groups, the presence of water adds enthalpic and entropic factors. Aggregation in aqueous environments is governed by a balance between solute–solute, solute–water, and water–water interactions (i.e., the self-aggregation energy of water).^1^ Even the inclusion of a single water molecule may substantially alter the structure of a monomer, for example, by restricting its conformational landscape.^2,3^ For instance, previous studies have shown that one water molecule is able to fold a β-strand into a γ-turn, induce the opening of an otherwise stable γ-turn in capped phenylalanine model peptides^4^ or alter the conformation of small bioactive molecules, such as the structure of aspartame.^5^

Despite the importance of solvation in molecular aggregation, only a few experimental studies have explored the impact of even a single water molecule on the structure of the aggregate.^6–8^ Here, we build upon those previous reports by presenting a spectroscopic and computational characterization of the aggregates formed by 2-phenylethanol (PEAL), 2-phenethylamine (PEA), and 2-phenethylthiol (PET) in jets.

## Results and Discussion

Previous studies^9–11^ have demonstrated that the three compounds tend to form very similar homo- and heterodimers, despite the different nature of their substituents, which results in hydrogen bonds of varying strength. The main difference among them lies in the number of conformers detected for each system.^10,11^ The addition of a water molecule significantly complicates spectroscopic analysis. For example, PEA exhibits a natural tendency toward fragmentation, as previously reported in the pioneering works by Simons’ group.^12^ Such a propensity appears to be enhanced by the presence of a single water molecule, which prevented the acquisition of spectroscopic data for the monohydrated PEA_2_ complex in this study.

Figure 1 presents the 2-color REMPI spectra of the four monohydrated dimers whose spectroscopy could be successfully recorded. Additional spectra obtained under different experimental conditions, as well as a comparison with the REMPI spectra of the corresponding dimers, are available in Figures S1–S4 (Supporting Information). Only the PEAL_2_-W_1_ spectrum was recorded on its own mass channel; the remaining monohydrates appeared as fragmentation products in the mass channels of their respective dimers. All four spectra fall within a narrow range of approximately 300 cm^–1^ and exhibit minimal vibrational activity. By tuning the probe laser to the transitions labeled with asterisks in Figure 1, the IDIR spectra shown in Figure 2 were recorded. The position of the OH, SH, and NH stretching bands is highly sensitive to the surrounding environment of the functional group, providing very valuable structural information. Furthermore, comparison with the simulated spectra obtained via normal-mode analysis of the computed structures for each aggregate enables structural assignment of the experimentally observed species (Figure 3 and Table 1).

**Figure 1:**
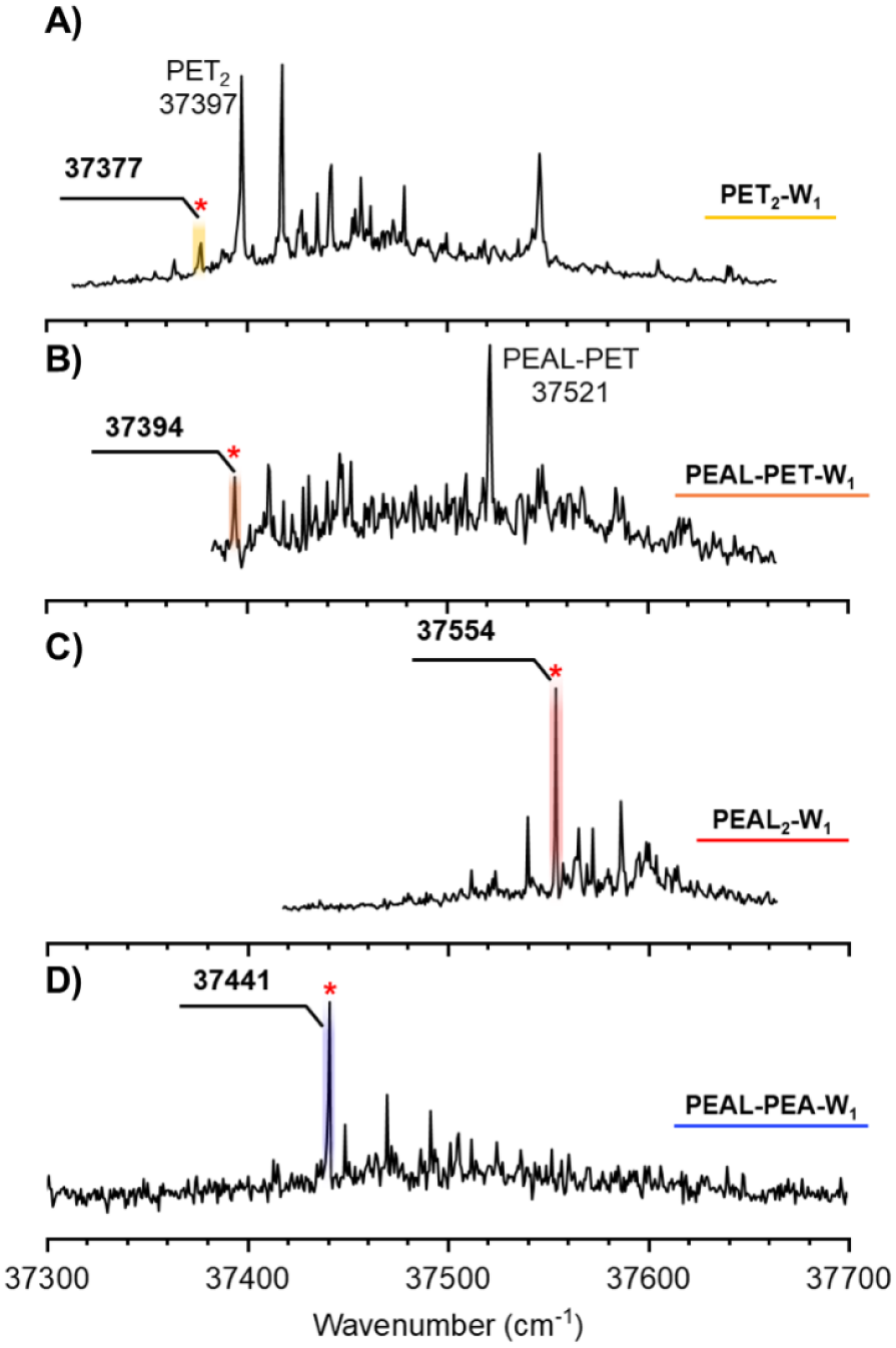
2-color REMPI spectra of the monohydrates of A) PET_2_, B) PEAL–PET, C) PEAL_2_, and D) PEAL–PEA. Only PEAL_2_-W_1_ was recorded on its own mass channel. The rest of the spectra were collected on the corresponding dimer’s mass channel. The bands used to record the IDIR spectra in Figure 2 are indicated with asterisks.

**Figure 2:**
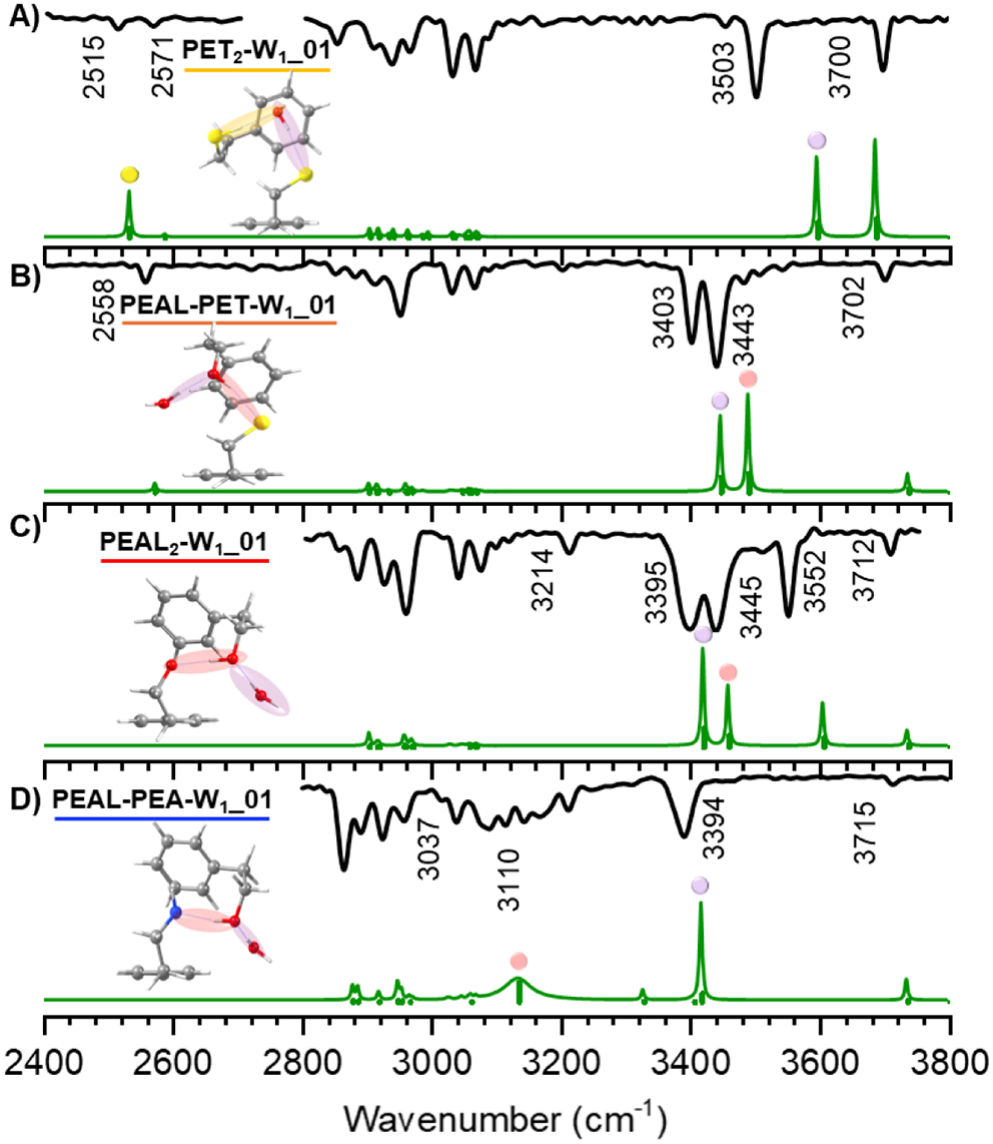
IDIR spectra of the monohydrates of A) PET_2_, B) PEAL–PET, C) PEAL_2_, and D) PEAL–PEA and a comparison with the spectra simulated using the calculations at the M06-2X/def2-TZVP level. A 0.951 correction factor was used to account for anharmonicity and deficiencies in the chosen DFT method. The color of the dots matches the color of the shaded bonds to which the vibrations correspond. Additional computed structures and the corresponding comparison between predicted and experimental spectra can be found in Figures S5 and S17 of the Supporting Information.

**Figure 3:**
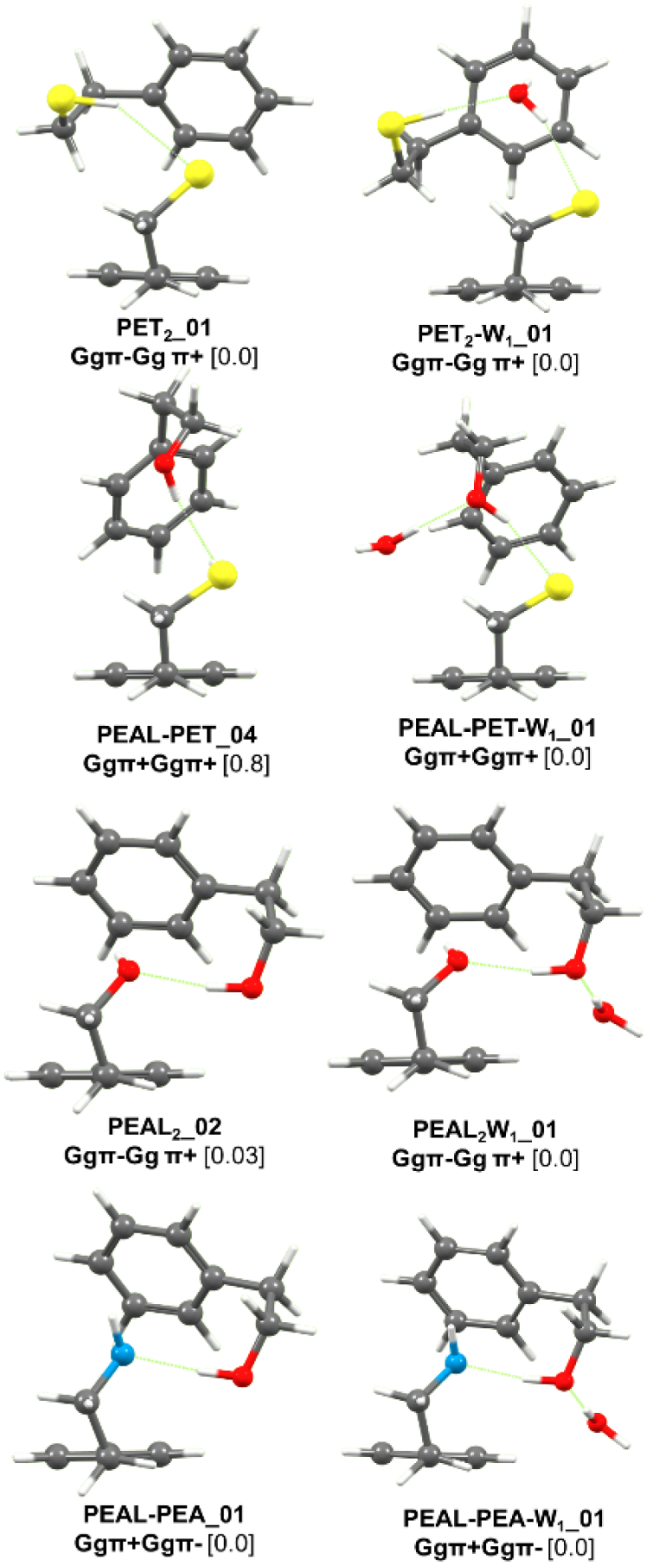
Comparison between the structures of the hydrated dimers. Calculations performed at the B3LYP-D3BJ/def2-TZVP level. Values represent the relative stability of each species in kJ·mol^–1^.

**Table 1:** Structural Parameters for the Structures Assigned in This Work Are Listed (Figure 3)­^a^

	PET_2_-W	PEALPET-W	PEAL_2_-W	PEALPEA-W
d(O_w_H···Y)	2.39	1.82	1.79	1.77
angle	162	170	173	174
ν(exp)	3503	3395	3395	3394
d(OH···Y)	2.08^b^	2.29	1.77	1.77
angle	170^b^	167	173	179
ν(exp)	2517	3403	3445	3110
d(XH···π)^c^	2.66	2.61	2.42	2.57
angle^d^	152	163	163	158
ν(exp)	2571	2558	3552	Not detected

a Distances in angs., angles in degrees, and stretching vibrations in wavenumbers.

b Corresponds to SH···OH_2_ distance and angle.

c Distance to the ring’s closest carbon atom.

d Angle with the ring’s closest carbon atom.

In all of the systems except PET_2_, the water adds to the dimer by forming a hydrogen bond with the proton-donor molecule. This behavior may be influenced by the kinetics of the aggregation process: if the homodimer forms first and the water molecule is added afterward, the energy released upon hydration may not be sufficient to induce isomerization, preventing the water molecule from adopting a central position. Nevertheless, all calculations indicate that the observed structures correspond to the most stable configurations, although in some cases the energy differences are small. Therefore, one can conclude that the final structures of the monohydrates studied here are primarily determined by energetic factors.

Assignment of the experimentally detected structures reveals that water can only dissociate the dimer’s hydrogen bond in PET_2_. In the rest of the aggregates, it accommodates as a proton-donor in the O_water_H···XH···X′H···π sequence (where X, X′ = N, O, S). The ability of water to break the SH···SH bond arises not only from the inherent weakness of this interaction^11^ but also from the formation of a hydrophilic core at the center of the aggregate. According to the NCI analysis (Figure 4), the SH···O interactions in PET_2_-W_1_ are still weak; indeed, they are the weakest hydrogen bonds among all four systems. However, this configuration allows the system to retain an SH···π interaction, while water establishes its own OH···π hydrogen bond.

**Figure 4:**
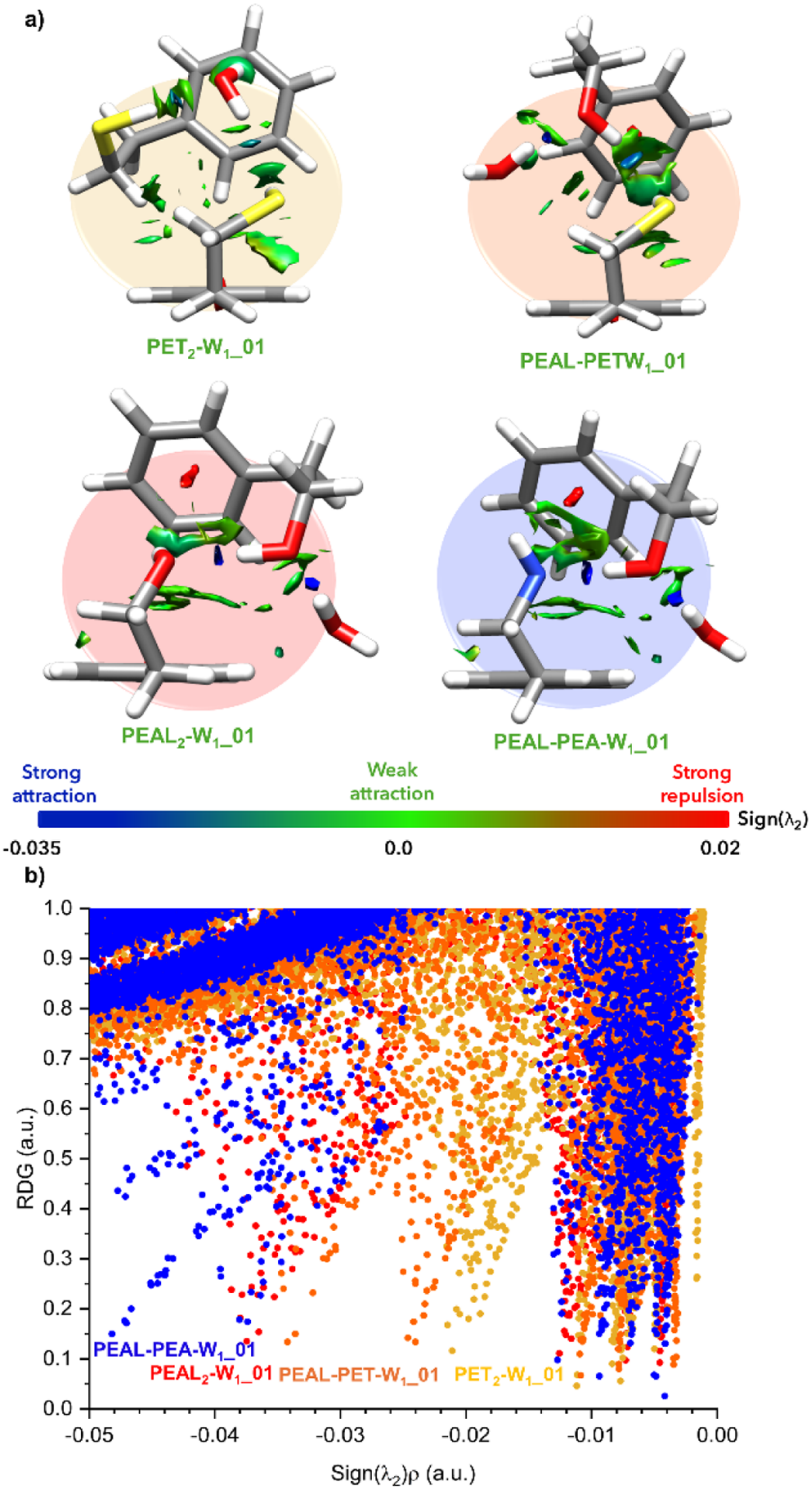
a) Surface representation of the NCIs and b) 2D-NCIplot analysis of the monohydrated dimers: PET_2_-W_1_, PEAL–PET-W_1_, PEAL_2_-W_1_, and PEAL–PEA-W_1_. Data were obtained from the calculations at the B3LYP-D3BJ/def2-TZVP level. An enlarged version of this figure has been placed in Supporting Information (Figure S19).

According to the NCI analysis, the hydrogen bonds involving water follow this strength sequence: O_w_H···S < SH···O_w_ < O_W_H···O_PEAL_(PEAL–PET-W_1_) < O_W_H···O_PEAL_(PEAL_2_-W_1_) ≲ O_W_H···O_PEAL_(PEAL–PEA-W_1_). This trend, illustrated in Figure 4b (see also Figures S18 and S19) using NCI analysis, aligns with expectations based on the electronegativity of the atoms involved.

A closer look at the remaining hydrogen bonds confirms that O_PEAL_H···N is the strongest interaction, consistent with the strong ability of N as a proton acceptor.^13^ The observed shift in the O_PEAL_H stretching band, 158 cm^–1^ from its position in the dimer and 539 cm^–1^ from the monomer (Figure 5), is a clear demonstration of such strength. This large shift moves the band into the CH stretching region, broadens it, and convolves it with the bands due to the CH stretches.

**Figure 5:**
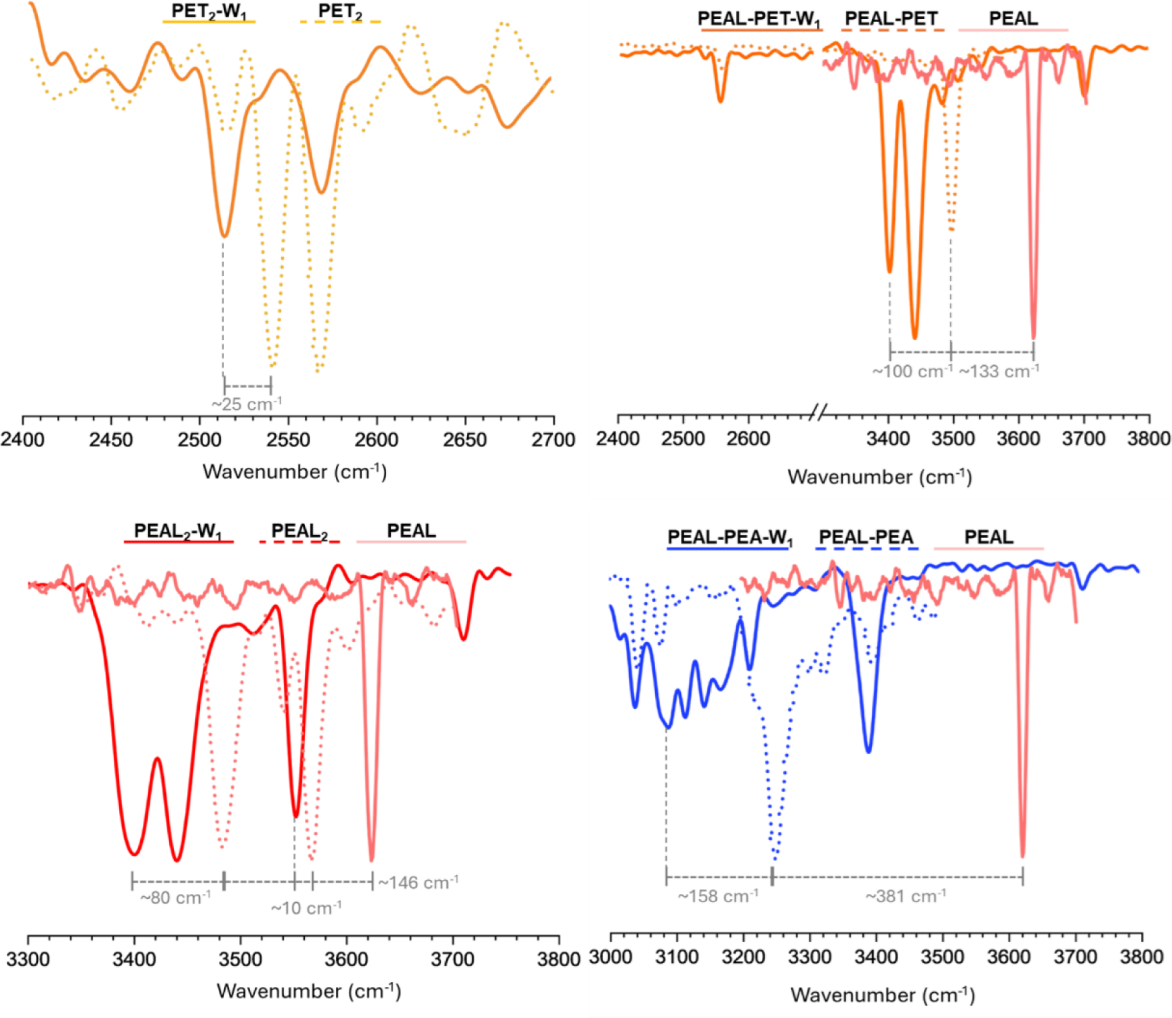
Comparison between the section of the mass-resolved IR spectra containing the X-H stretches (X = O, N, or S) of the monohydrated dimers studied in this work with the corresponding vibrations in the dimers and monomers. The shifts in the PEAL OH stretch are indicated.

In contrast, the stretching band of O_PEAL_H in PEAL–PET-W_1_ shifts by 100 cm^–1^ upon hydration, yielding a total shift of 233 cm^–1^ relative to that of the bare molecule. Interestingly, this is approximately the shift observed for the O_PEAL_H···O_PEAL_ interaction in PEAL_2_-W_1_, which, according to the NCI analysis in Figure 4, is ∼50% stronger (−32.6 vs 19.7 cm^–1^, see also Table S2). It is generally accepted that there is a direct correlation between hydrogen bond strength and the shift in the corresponding stretching vibration.^14^ Attempts to express this relationship in the form of a mathematical equation date back to the work by Murthy and Rao,^15,16^ who reported different correlations between spectral shifts and binding energies (Δ*H*), depending on the acceptor atom.^16^ For the systems analyzed in this study, such a relationship exists, although it differs between PEAL_2_ and PEAL_2_-W_1_ and the rest of the aggregates (Figure S19). The shift induced by hydrogen bond formation is primarily due to the transfer of electronic density from the nonbonding orbitals of the acceptor atom to the σ* orbital of the donor, weakening and elongating the X-H bond. However, in systems dominated by London dispersion forces, the elongation of the X-H bond may be minimal or even absent. Such is the case of the improper, blue-shifted hydrogen bonds.^17^

The different correlations observed in Figure S20 may be explained by the relative contribution of charge transfer and electrostatic interactions to the hydrogen bond. In the case of OH···S interactions, the large size of the 3s/3p orbitals of the acceptor sulfur atom may facilitate the overlap with the σ* orbital of the donor OH, resulting in a higher spectral shift/binding energy ratio. This is consistent with the findings by Biswal and Wategaonkar^18^ regarding NH···O/NH···S hydrogen bonds. In the case of OH···N interactions, previous studies attempting to correlate OH stretch shifts with properties such as proton affinity have revealed distinct trends: dimers with OH···O interactions, such as phenol···water and phenol···methanol, behave differently from those with OH···N interactions, such as phenol···ammonia or phenol···methylamine.^19,20^ These observations suggest that oxygen and nitrogen acceptors produce different spectral shifts. Surprisingly, however, a correlation is found between the shifts produced by OH···S and OH···N interactions, despite the markedly different nature of these hydrogen bonds, an observation that merits further investigation, but it may be related to the larger electron-donating ability of N compared to O. Previous studies^21^ found a correlation between the shift and different computed parameters (electron volume, electron density, etc.), but always taking into account the nature of the acceptor atom.

An intriguing aspect of this study is the effect of water on the structure of the dimers. While water was able to insert between the two monomers in PET_2_, it caused only modest structural changes in the other dimers. Molecules with OH groups tend to form cyclic structures up to the hexamer,^8,22–27^ with very few exceptions, mostly at the trimer level.^10,23^ Previous studies on PEAL_2–4_ ^10^ and benzyl alcohol up to the tetramer^23^ have shown that only the tetramers of this type of system start forming cyclic structures, while trimers prefer OH···OH···OH···π hydrogen bond networks. Cyclic trimeric hydrogen bonds often force bond angles away from their optimal value, inducing strain, whereas interactions with the π-electron cloud offer flexibility to the molecules to accommodate and optimize OH···O interactions. Thus, in the systems studied here, there is a competition between water integrating into the hydrogen bond network to form cyclic structures and simply elongating the preexisting network. Experimentally, no cyclic structures were detected. Although such isomers were found computationally, they are slightly less stable than the assigned structures (see Figures S5–S12).

Spectroscopic studies of dimer solvation are rare. In the case of the hydrated propofol dimer,^7^ the accessibility of the solvation site likely facilitated the formation of a cyclic hydrogen bond network, resulting in a structure similar to those observed in phenol trimers, water trimers, and other alcohol trimers.^27,28^ The hydrated aniline dimer represents, perhaps, the most extreme case of dimer reorganization: while the dimer forms a symmetric structure with the two amino groups establishing NH_2_···π interactions with the aromatic ring of the partner molecule,^29^ the addition of a water molecule forces the system to adopt a cyclic hydrogen bond network.^8^ Therefore, the absence of cyclic hydrogen bond structures in the systems studied here may be due to a combination of lower thermodynamic stability and limited accessibility to suitable solvation sites.

Another open question is why water did not insert into the dimer’s hydrogen bond, except in the case of PET_2_. For example, in the solvation of glycoaldehyde dimer, water inserts into one of the two symmetric hydrogen bonds formed by the glycoaldehyde molecules.^6^ In fact, the authors of ref. 6 focused their study on water’s ability to reshape hydrogen bond networks. The strength of the hydrogen bond between glycoaldehyde molecules is like that of O_PEAL_H···O_PEAL_ and exhibits flexibility similar to that of the systems studied here. Nevertheless, the strength of the hydrogen bond does not preclude water from inserting into the OH···O interaction in formic acid^30^ or in benzoic acid dimer,^31^ so it seems that the strength alone is not the dominant factor in the competition between insertion and addition. Perhaps the main difference between glycoaldehyde dimer, formic acid dimer, and, for example, PEAL_2_ may lie in the accessibility of the insertion point or in the kinetics of the aggregation process. Understanding the mechanics of the process truly deserves further investigation.

A final note of caution when interpreting the data obtained in the gas phase: the ability of water to incorporate between the two PET molecules may suggest a higher solubility of PET compared with PEA or PEAL. However, solubility also depends on water’s self-aggregation energy, which must be balanced against water–solute interactions. Since PET-water interaction is the weakest among the systems studied, this becomes the key parameter when comparing gas-phase behavior with solubility data. An example of this behavior is the structure of the benzene-water aggregates recently reported by Steber et al.:^32^ while in solution, water forces benzene molecules to stack and group together to minimize their contact with the water molecules; in the gas-phase, in aggregates of limited size, water inserts between the benzene molecules and conditions their relative position. Even in this highly hydrophobic environment, water is able to direct the aggregation process and impose its preferences on the final shape of the cluster.

In summary, this work contributes to the limited body of data on the solvation of organic dimers. By evaluating the structures, interaction energies, and spectroscopic shifts, we gained insight into the nature of hydrogen bonding and its correlation with the spectroscopic observations. Interestingly, despite the wide range of hydrogen bond strengths among the systems studied, all except PET_2_-W_1_ form similar structures, with water adding to the preexisting hydrogen bond network and disregarding other stable isomers that could theoretically form. Several open questions remain, highlighting the need for new and diverse data on the solvation of molecular aggregates.

## Methods

An enlarged version of the methods section can be found in the Supporting Information. Briefly, the experiments were carried out in an in-house-designed time-of-flight mass spectrometer. Cooling was achieved using a pulsed valve (Jordan Inc.) operated at 10 Hz and using He, Ne, and/or Ar as backing gases. The samples were heated up to 343 K to achieve enough vapor to record the spectra. A set of lasers (Quantel Brilliant B + Fine adjustment and Quantel Qsmart 850 + Qscan, LaserVision OPO system) was used to record REMPI and/or IDIR spectra. Calculations were carried out using Schrödinger software^33^ to explore the potential energy surface of the aggregates. The isomers obtained were grouped into families of similar structures and interactions. Some selected structures representative of the families found were subjected to full optimization at the B3LYP/def2-TZVP level using Gaussian 16.^34^ To confirm the assignment of the IR spectra, a second round of optimizations was carried out at the M06-2X/def2-TZVP level on the most stable structures (see Supporting Information).

## Supplementary Material



## Notes

Additional methods description, additional computed structures, comparison between experimental IR spectra and those simulated using the computational data (PDF)
