# Supplementary material for: Understanding the Influence of a Water Molecule in the Structure of a Dimer
Source: ACS Omega. 2026 Feb 6;11(7):12371–7. doi: 10.1021/acsomega.5c11915 (PMC12947019; doi:10.1021/acsomega.5c11915)
Supplement: Supplementary file 1 [file ao5c11915_si_001.pdf]

Supporting Information for:

Understanding the influence of a water molecule in the structure of a dimer

Fernando Torres-Hernández, Paúl Pinillos, Ander Camiruaga, Imanol Usabiaga and José A. Fernández.

Department of Physical Chemistry, Faculty of Science and Technology, University of the Basque Country (UPV/EHU), Barrio Sarriena s/n, Leioa 48940, Spain.

Experimental and computational methods

Experimental methods. A detailed description of the experimental set up may be found in previous publications.[1,2] Briefly, the experimental set up consists of an in-house designed linear time-of-flight (ToF) mass spectrometer; two systems of Nd/YAG laser + dye laser + doubling unit (Quantel Brilliant B + Fine adjustment and Quantel Qsmart 850 + Qscan) and an OPO system (LaserVision) pumped by a Nd/YAG laser (Continuum mod Surelite), together with the required electronics for control and signal acquisition.

The sample (2-phenylethanethiol, 2-phenylethanol and 2-phenylethylamine, and/or a mixture of them with water) was deposited in a sample holder and inserted in the gas line feeding a pulsed valve (Jordan Inc.) attached to the ionization chamber of an in-house designed mass spectrometer. At each aperture of the valve, operated at 10 Hz, a supersonic expansion was created, producing an adiabatic cooling of the molecules down to 2-5 K of rotational temperature and 50 – 100 K of vibrational temperature. Under such conditions, the molecules aggregate. Either He or Ne were used as buffer gas at typical pressures between ~2-3 bar. The species populating the beam were excited with UV photons that first excited the $S_1 \leftarrow S_0$ electronic transition and then ionize the molecules. The electric field, created by a pair of extraction/repulsion plates with 400 V of voltage difference between them, sent the ions towards the detector, accelerating them according to their charge-to-mass ratio. Before reaching the field-free flight path, the ions were further accelerated by an acceleration plate. During the travel through the field-free region of the mass spectrometer, the ions segregate according to their mass, arriving to the detector at different times. The detector consisted of a pair of microchannel plates that produced a ~4 mV pulse per ion. The signal generated by the ions was collected with the aid of a digital oscilloscope (Tektronix TDS 3032), integrated and routed to the same computer that controls the whole experiment. Synchronization between the lasers, valve and oscilloscope was handled using three pulse generators (SRS 645, Standford Research Systems).

In the 2-color resonance enhanced multiphoton ionization (REMPI) experiments, the excitation laser was scanned through the region of interest, while the total ion current in a given mass-channel was recorded. In this way, the mass-resolved electronic excitation spectrum of the molecules of interest was recorded, with ~0.1 cm^{-1} spectral resolution. In the IDIRS (ion-dip infrared spectroscopy) experiments, an additional IR laser beam was used, fired ca. 400 ns prior to the UV lasers. To record the spectra, the IR laser was scanned through the SH, NH and OH stretching mode region, while the excitation laser was kept tuned to an electronic transition of the species of interest. When the IR photons were resonant with a vibrational transition of the species probed by the UV laser, a dip in the ion signal was produced. In this way, the isomer-specific mass-resolved IR spectra of the species in the beam was recorded.

Computational methods. The conformational search was performed using molecular mechanics,[3,4] covering a large dataset of initial structures. All starting geometries were optimized with B3LYP[5] density-functional theory method, incorporating D3[6] empirical dispersion corrections (Becke-Johnson[7] damping function). The best candidates for the assignment of the experimental spectra were also optimized using M06-2X,[8] as an extra check of the validity of the assignments. In all cases, the def2-TZVP[9] basis set was used. Frequency calculations were performed using the harmonic approximation at the same level of theory. A correction factor of 0.951 was introduced to account for anharmonicity and other minor corrections. Such factor was deduced from the comparison between harmonic frequencies and experimental IRID spectra for the monomers and later refined using the data for the dimers. The interaction energies were calculated considering the basis set superposition errors (BSSE).[10] All calculations were performed using Gaussian 16,[11] selecting tight conditions for geometry optimizations. The presence of non-covalent interactions was analyzed using the NCIplot method,[12,13] based on a reduced gradient of electronic density. Calculation of the interaction strength of individual hydrogen bonds was done following the methodology on Ref 14. Briefly, the hydrogen bond binding energy (HBBE) can be calculated using two equations based on electron density at the bond critical point (BCP) associated with the hydrogen bond:

$$BE \approx -223.08\, x\, \rho(\mathbf{r}\text{BCP}) + 0.7423 \text{ (for neutral H−bond)}$$

$$BE \approx -332.34\, x\, \rho(\mathbf{r}\text{BCP}) - 1.0661 \text{ (for charged H−bond)}$$

In this case, the first equation was employed to estimate the HBBE of the hydrogen bonds present in the aggregates. The individual H-bond contributions were then calculated relative to the total binding energy.

Figure S1. 2C-REMPI spectrum recorded on PET_2 mass channel. A single band was identified as belonging to PET_2-W_1. Spectra recorded using 2 bars of Ne. The second color was tuned at 32786 cm^{-1}.

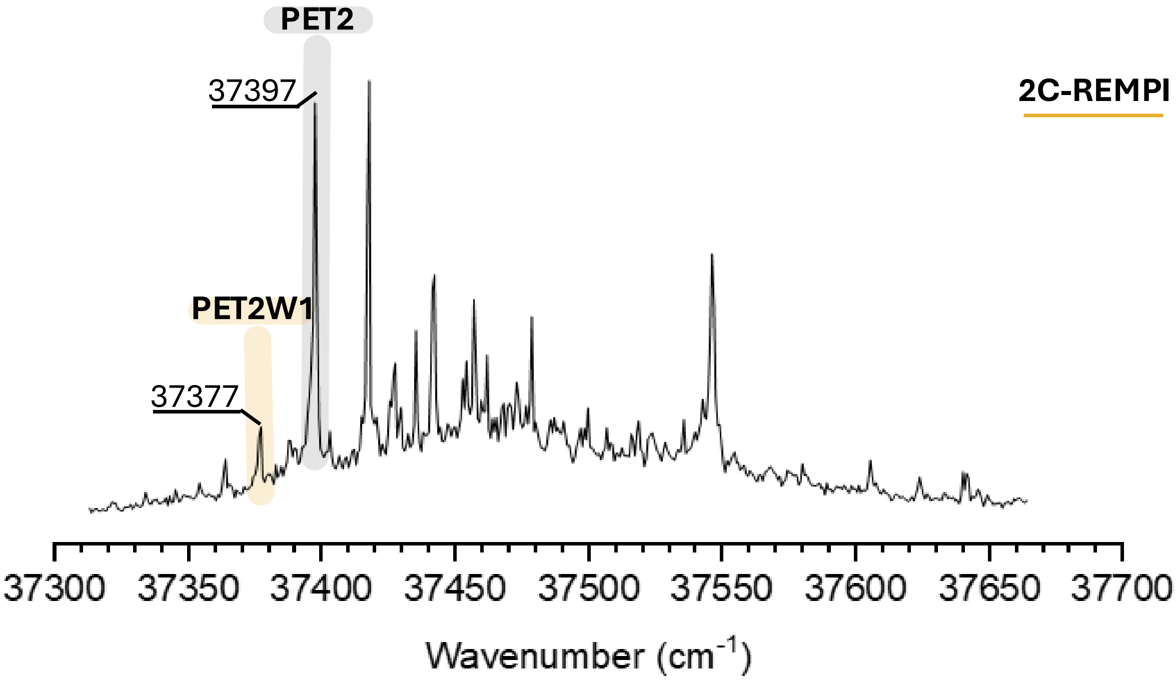

Figure S2. 1C- and 2C-REMPI of PEAL-PET with the band belonging to fragmentation from PEAL-PET-W_1 highlighted. Spectra recorded using 2 bars of Ne. The second color was tuned at 32786 cm^{-1} in the 2C-REMPI experiment.

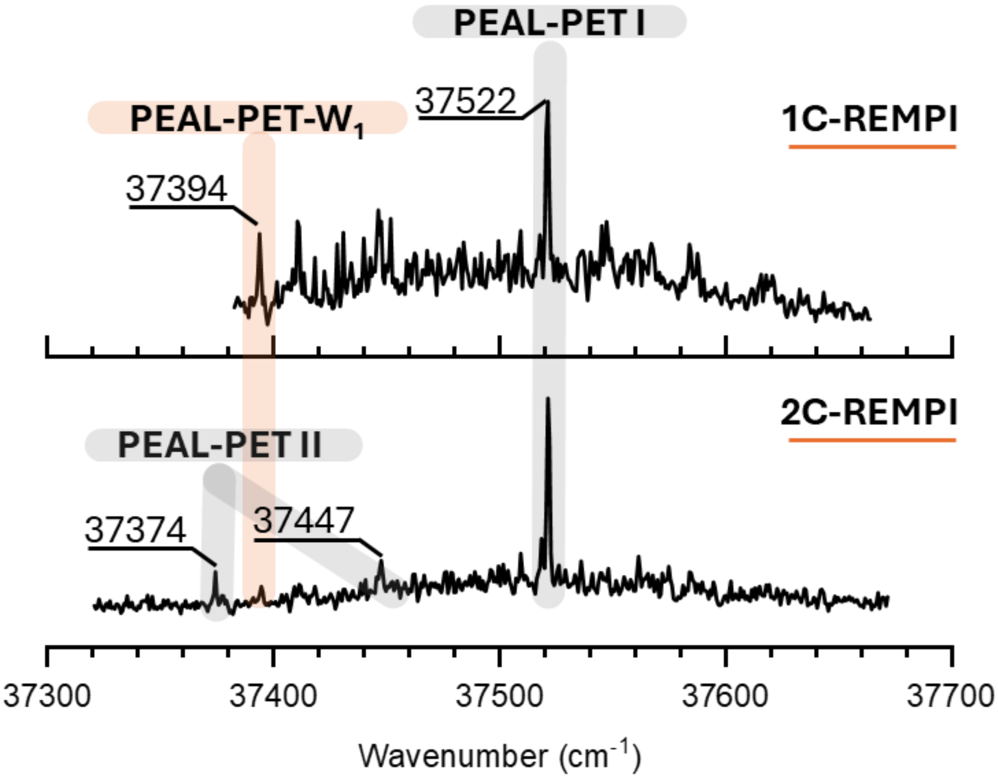

Figure S3. 2C-REMPI spectra of $PEAL_2$ and $PEAL_2$-W_1, recorded in their own mass-channels. Spectra recorded using 2 bars of Ne with the second color tuned at 35087 cm^{-1}.

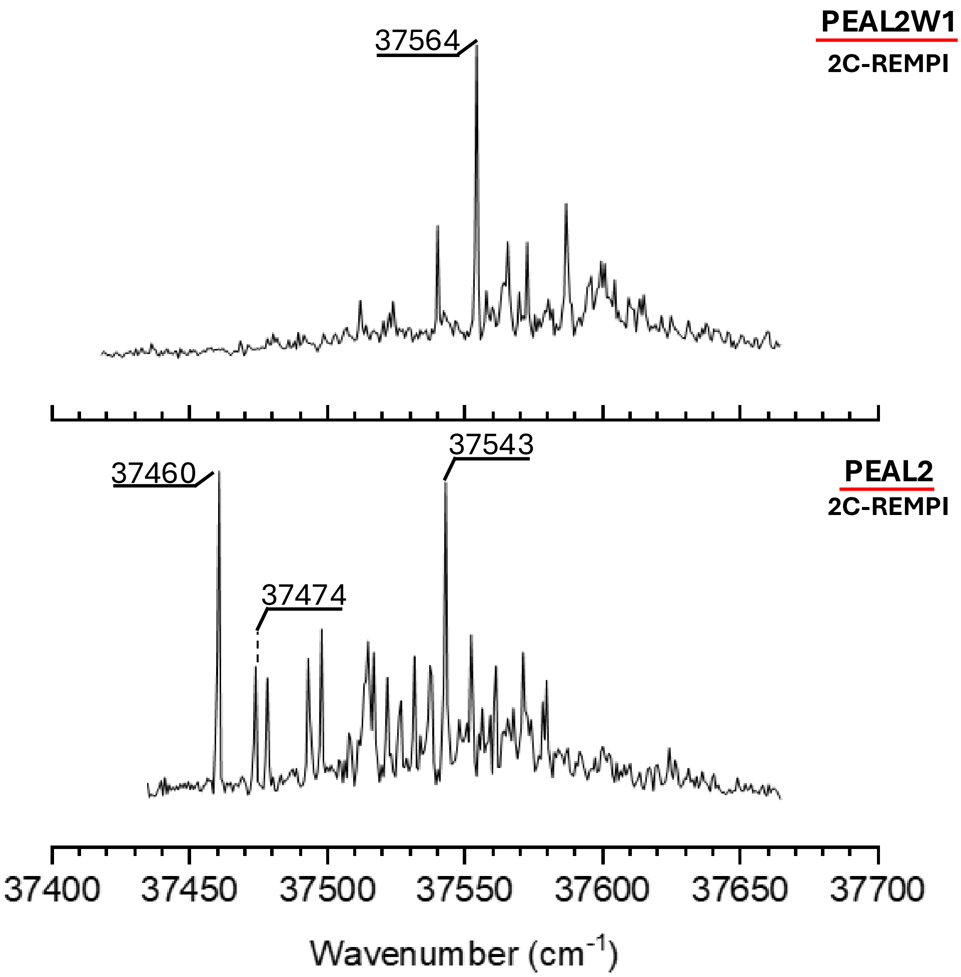

Figure S4. a) 1C- and 2C-REMPI spectra of PEAL-PET heterodimer and PEAL-PET-W_1 recorded in PEAL-PET mass channel using 2 bars of different carrier gases: He, 10% of Ar in He and Ne. b) Detail of the origin band region in the 2C-REMPI spectrum recorded with He. Ionization laser was set at 32258 cm^{-1}.

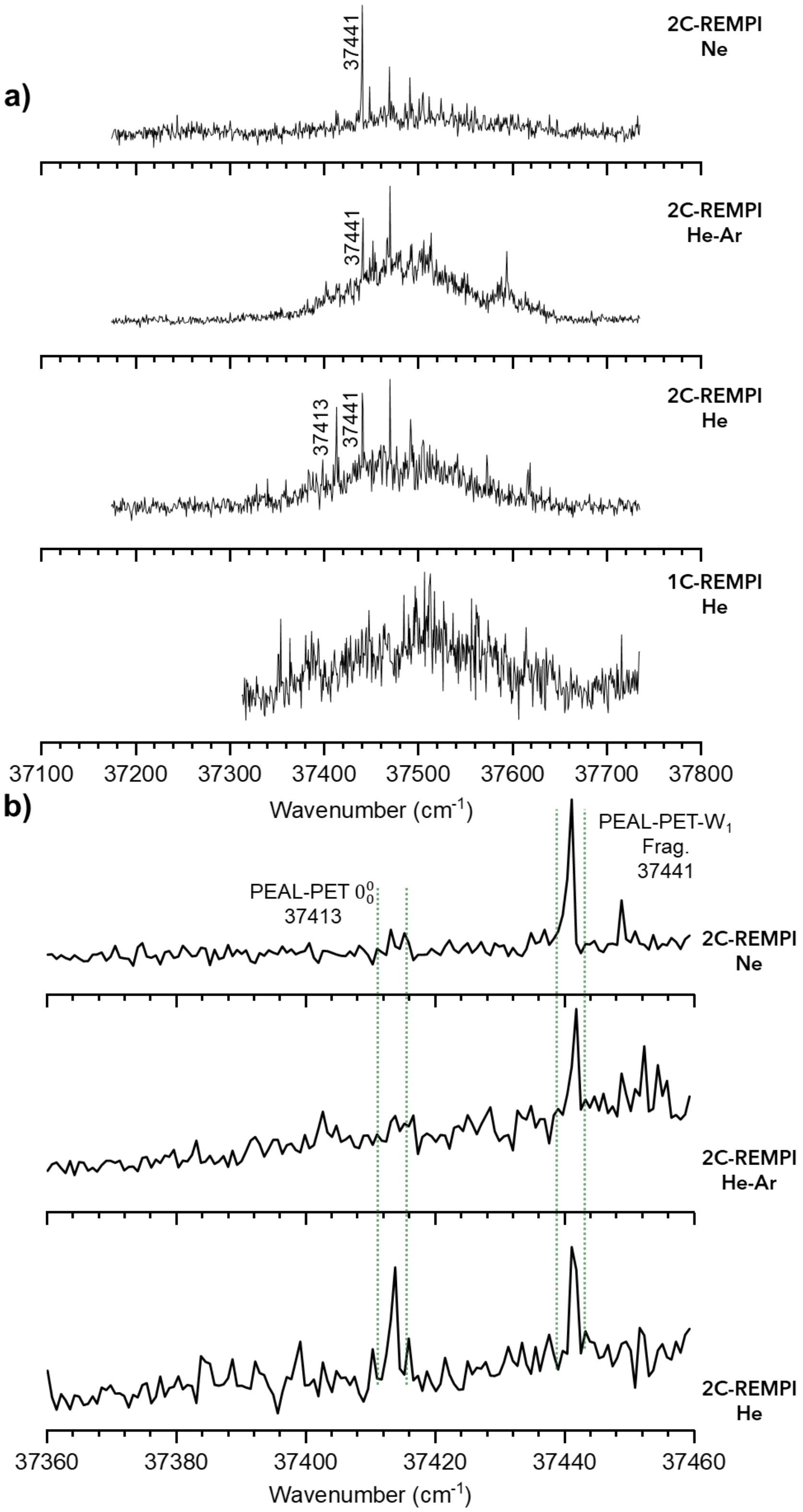

Figure S5. Representative structures of the PET_2-W_1 families, categorized by their interactions and relative energies. All structures were optimized using the B3LYP-D3BJ/def2-TZVP level of theory, with energies provided in kJ/mol.

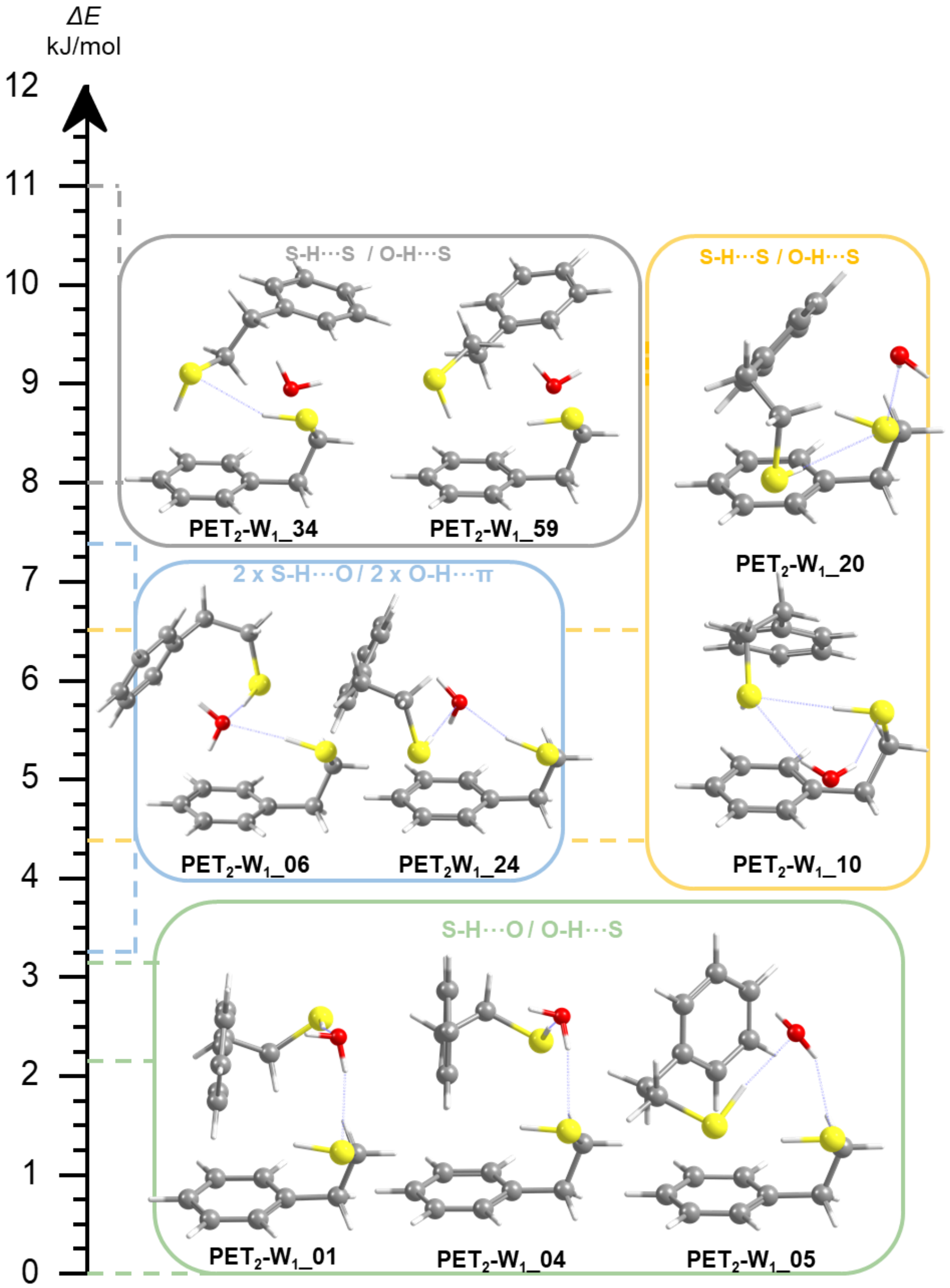

Figure S6. IDIR spectrum of the observed isomer of PET_2-W_1 compared to the simulated IR spectra. Simulations were calculated at the B3LYP-D3BJ/def2-TZVP level. A scaling factor of 0.963 was used for both OH and CH regions.

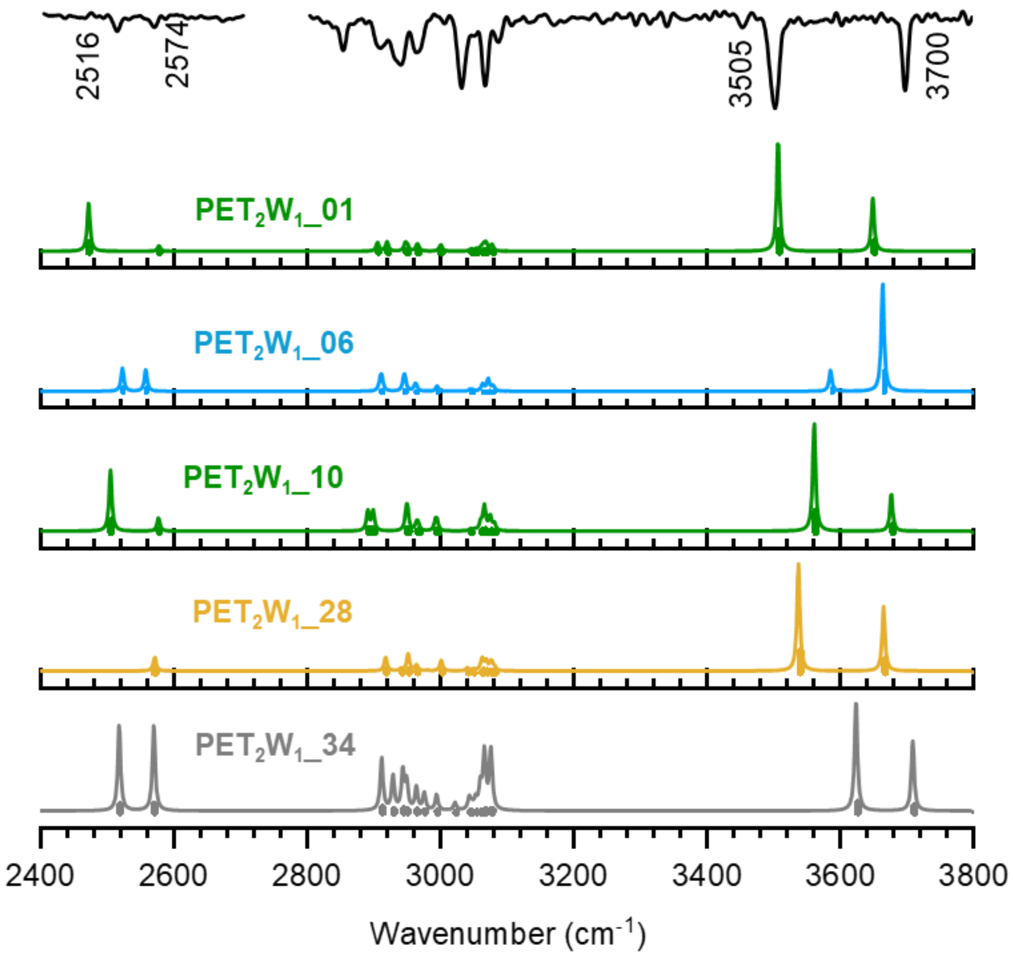

Figure S7. Representative structures of PEAL-PET-W_1 families, categorized by their interactions and relative energies. All structures were optimized using the B3LYP-D3BJ/def2-TZVP level of theory, with energies provided in kJ/mol.

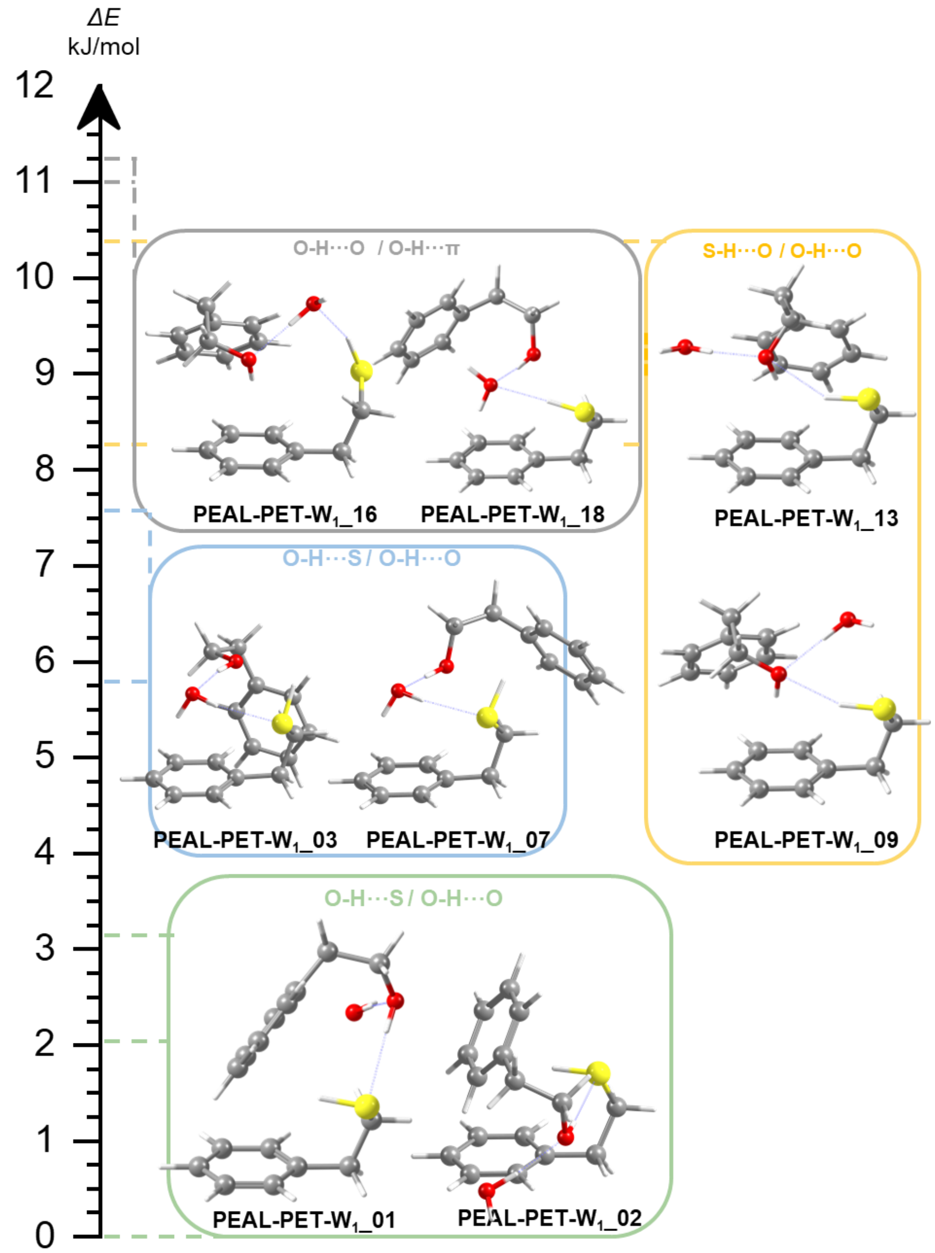

Figure S8. IDIR spectrum of the observed isomer for PEAL-PET-W_1 compared to the frequency predictions. Spectra simulations were calculated at the B3LYP-D3BJ/def2-TZVP level. Scaling factors of 0.963 were used for O-H, C-H and S-H region.

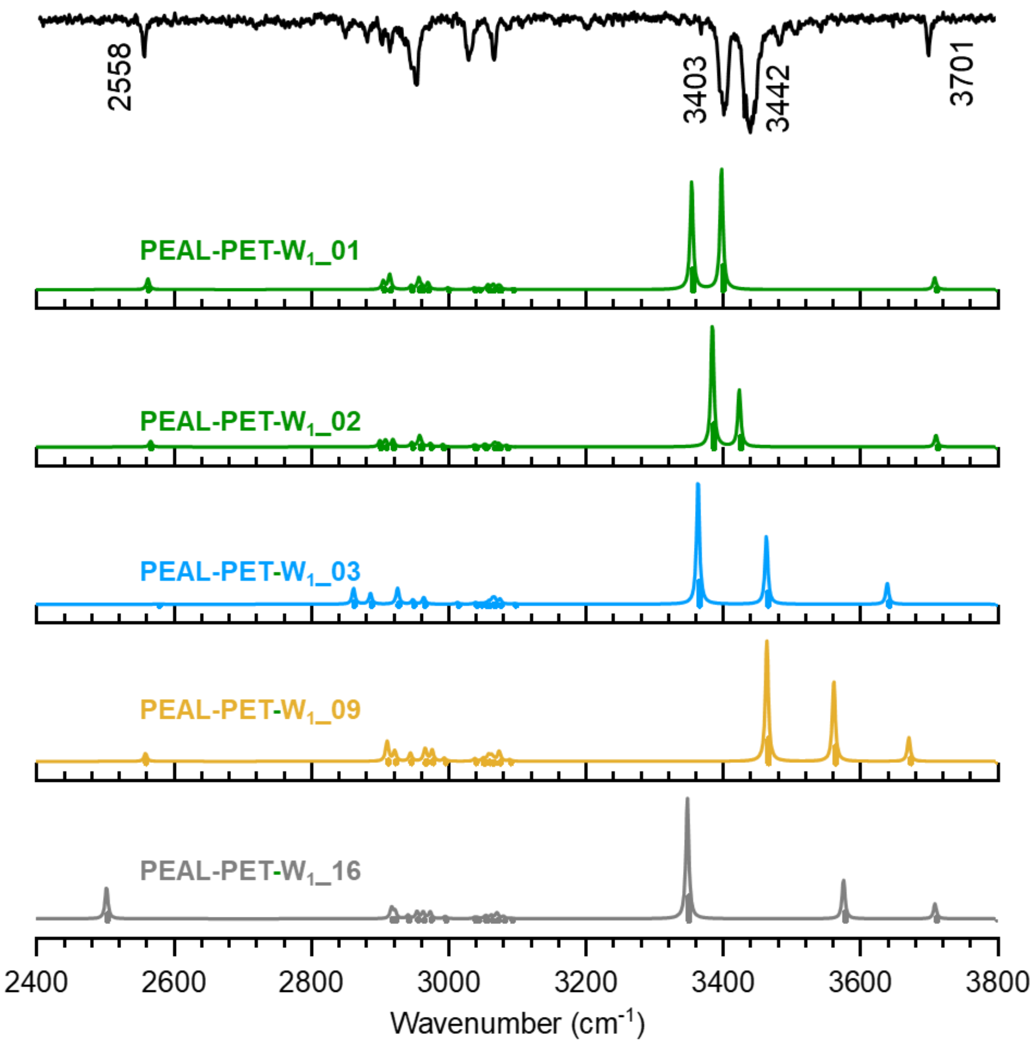

Figure S9. Families of monohydrated $PEAL_2$ according to their interactions and relative stability. The calculations were performed at B3LYP-D3BJ/def2-TZVP level and the enegies are provided in kJ/mol.

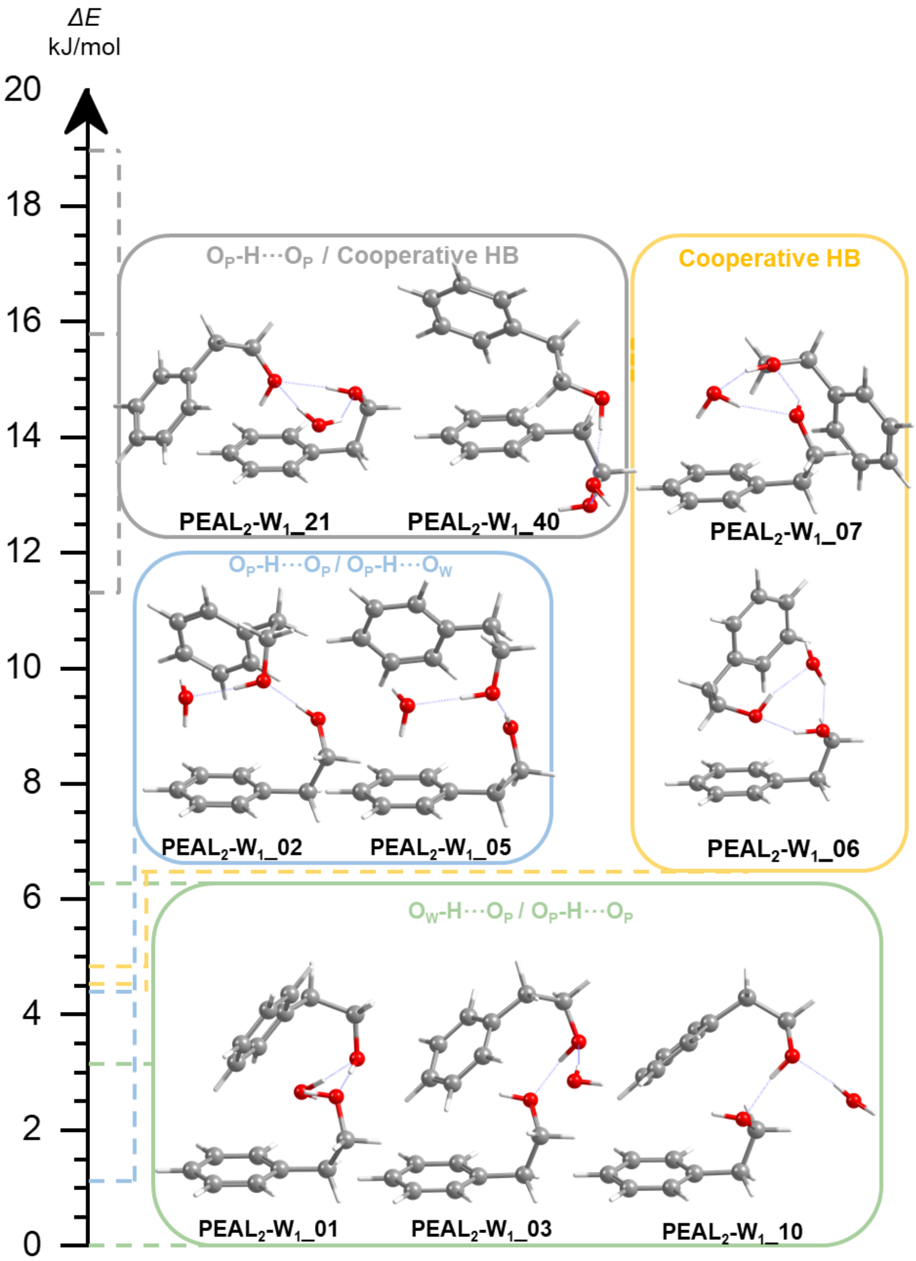

Figure S10. IDIR spectrum of the single observed isomer of $PEAL_2$-W_1 compared to the simulated IR spectra. Simulations were calculated at the B3LYP-D3BJ/def2-TZVP level. A scaling factor of 0.963 was used for OH and CH regions.

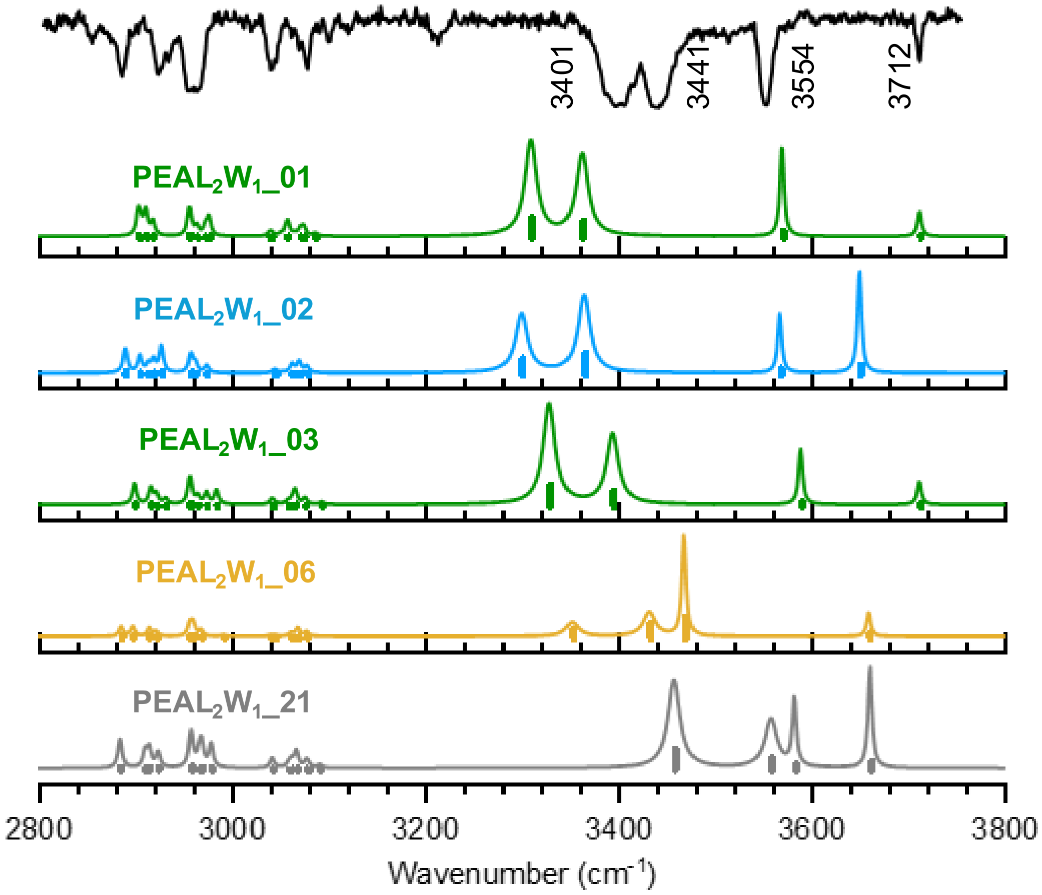

Figure S11. Representative structures of PEAPEALW1 families, categorized by their interactions and relative energies. All structures were optimized using the B3LYP-D3BJ/def2-TZVP level of theory, with energies provided in kJ/mol.

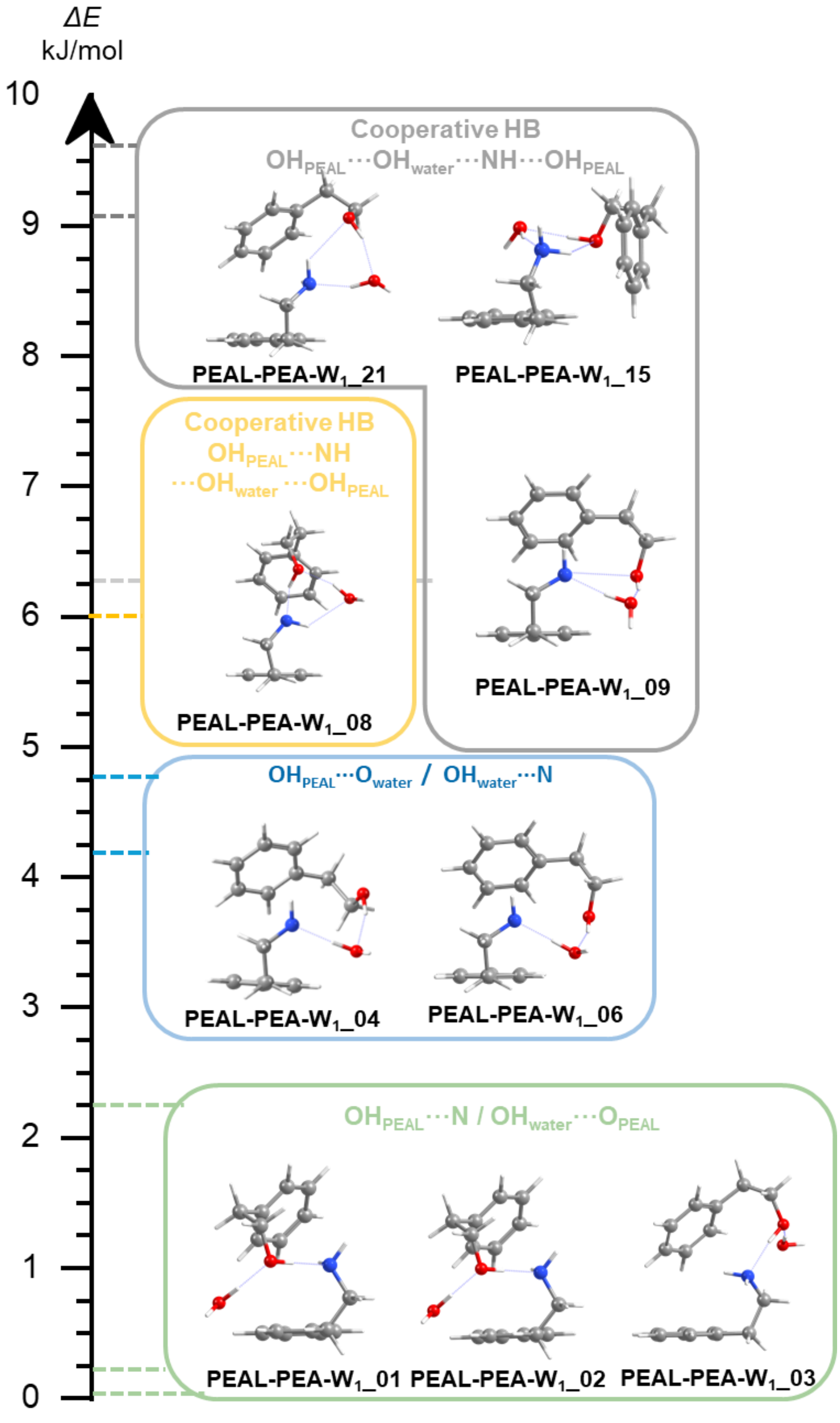

Figure S12. IDIR spectrum of the single observed isomer of PEAL-PEA-W_1 compared to the simulated IR spectra. Simulations were calculated at the B3LYP-D3BJ/def2-TZVP level. A scaling factor of 0.963 was used for OH, NH and CH regions.

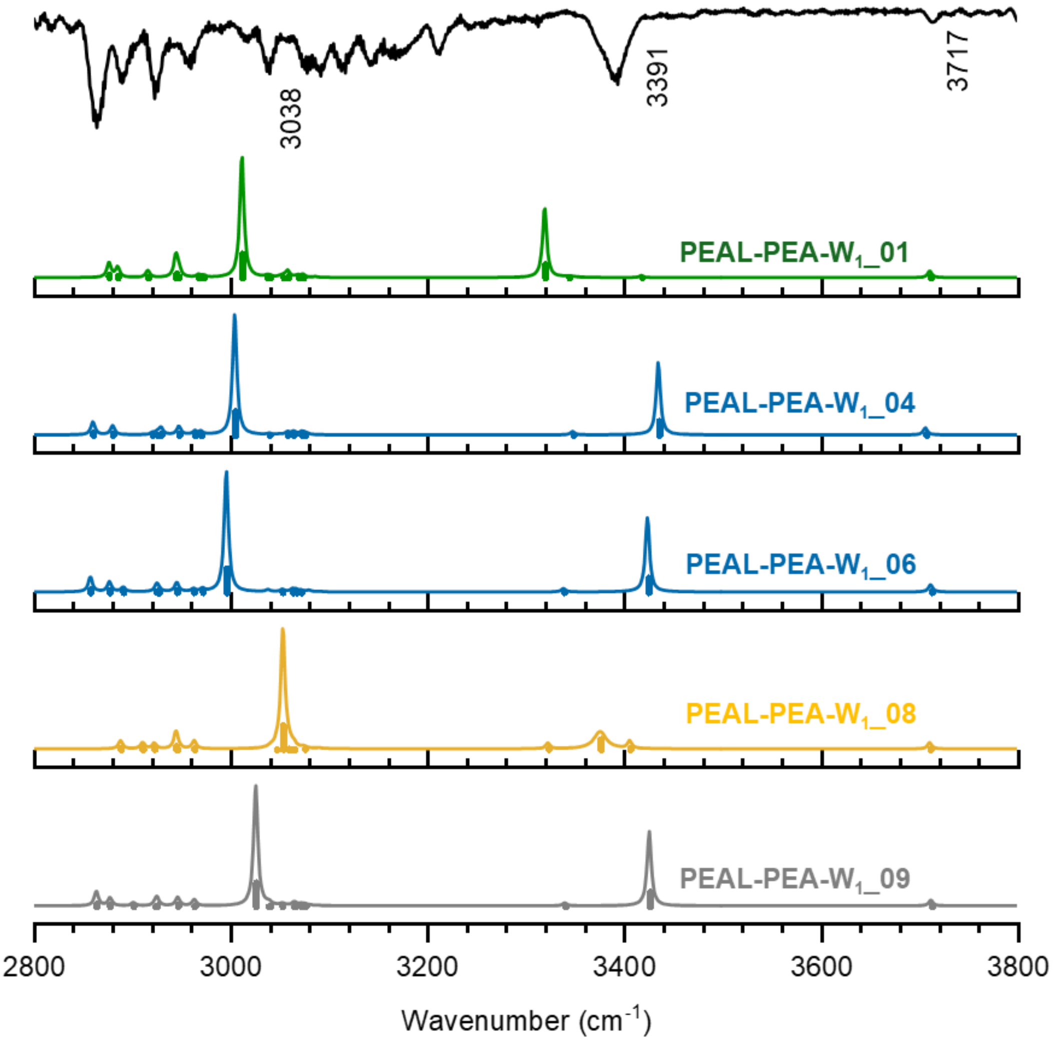

Figure S13. Comparison between the IDIR spectra of the monohydrates of PET_2, $PEAL_2$, PETPEAL, and PEAPEAL

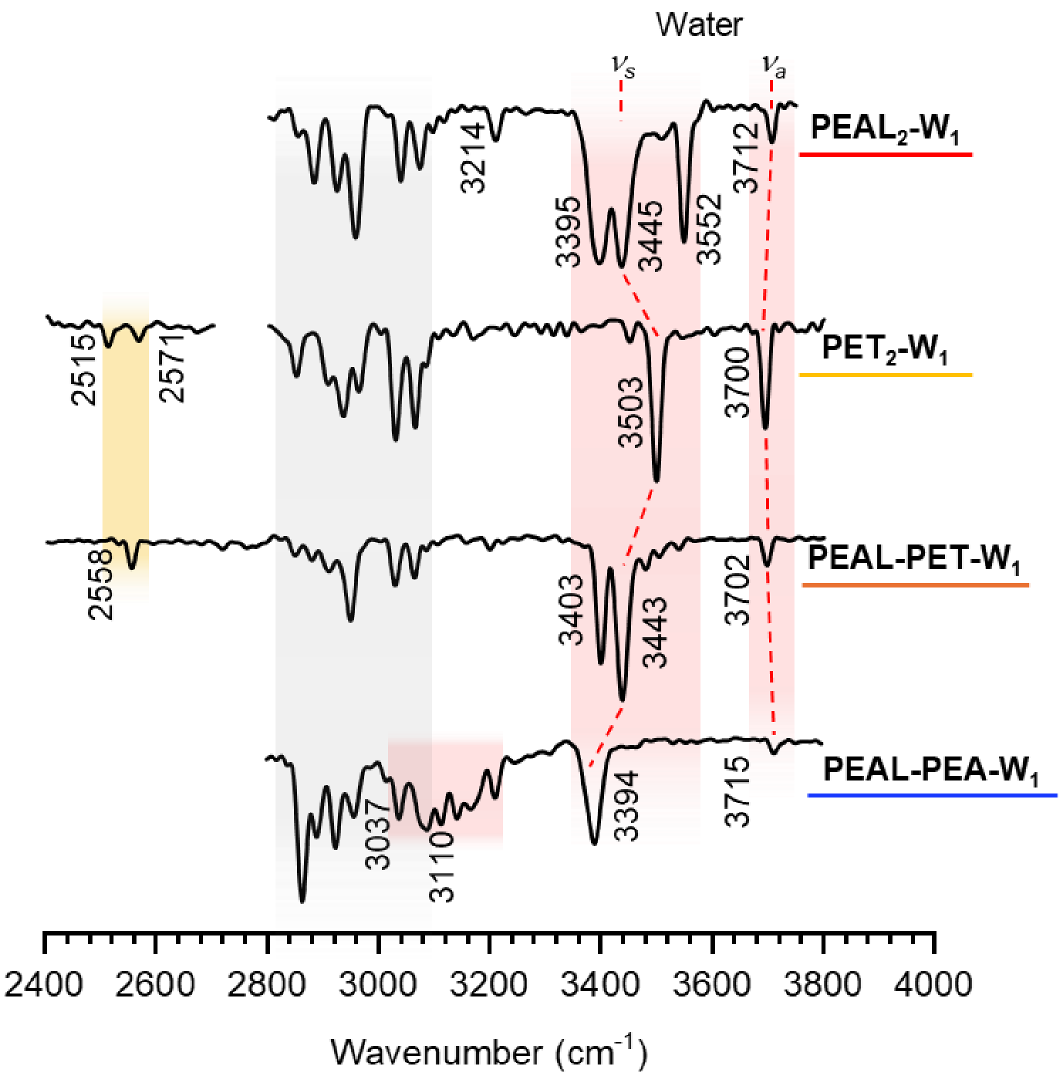

Figure S14. IDIR spectrum of the observed isomer of PET_2-W_1 compared to the simulated IR spectra at the B3LYP-D3BJ/def2-TZVP and M06-2X/def2-TZVP levels. A scaling factor of 0.963 was used for B3LYP-D3BJ/def2-TZVP and 0.951 for M06-2X/def2-TZVP.

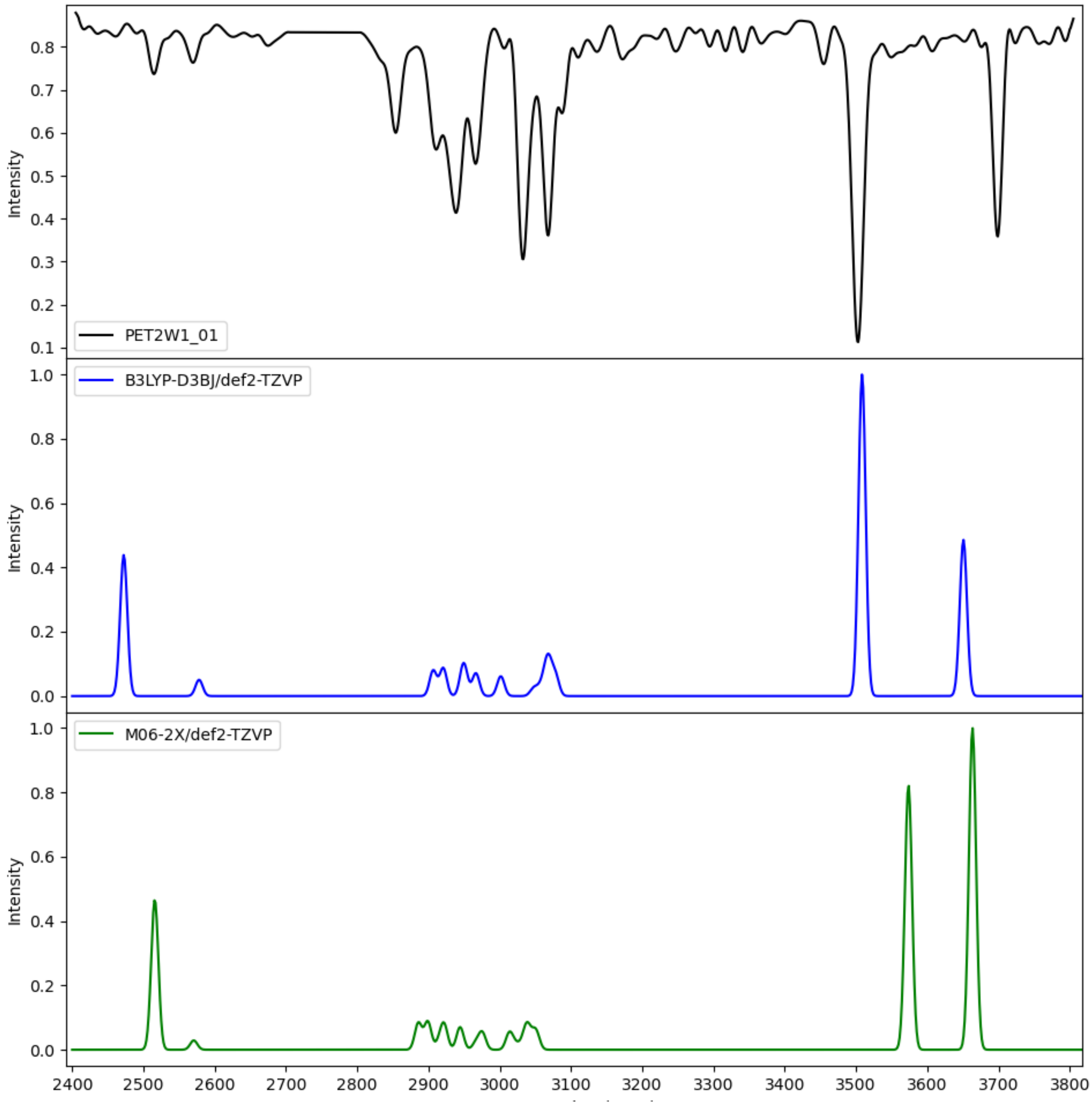

Figure S15. IDIR spectrum of the observed isomer of PEAL-PET-W_1 compared to the simulated IR spectra at the B3LYP-D3BJ/def2-TZVP and M06-2X/def2-TZVP levels. A scaling factor of 0.963 was used for B3LYP-D3BJ/def2-TZVP and 0.951 for M06-2X/def2-TZVP.

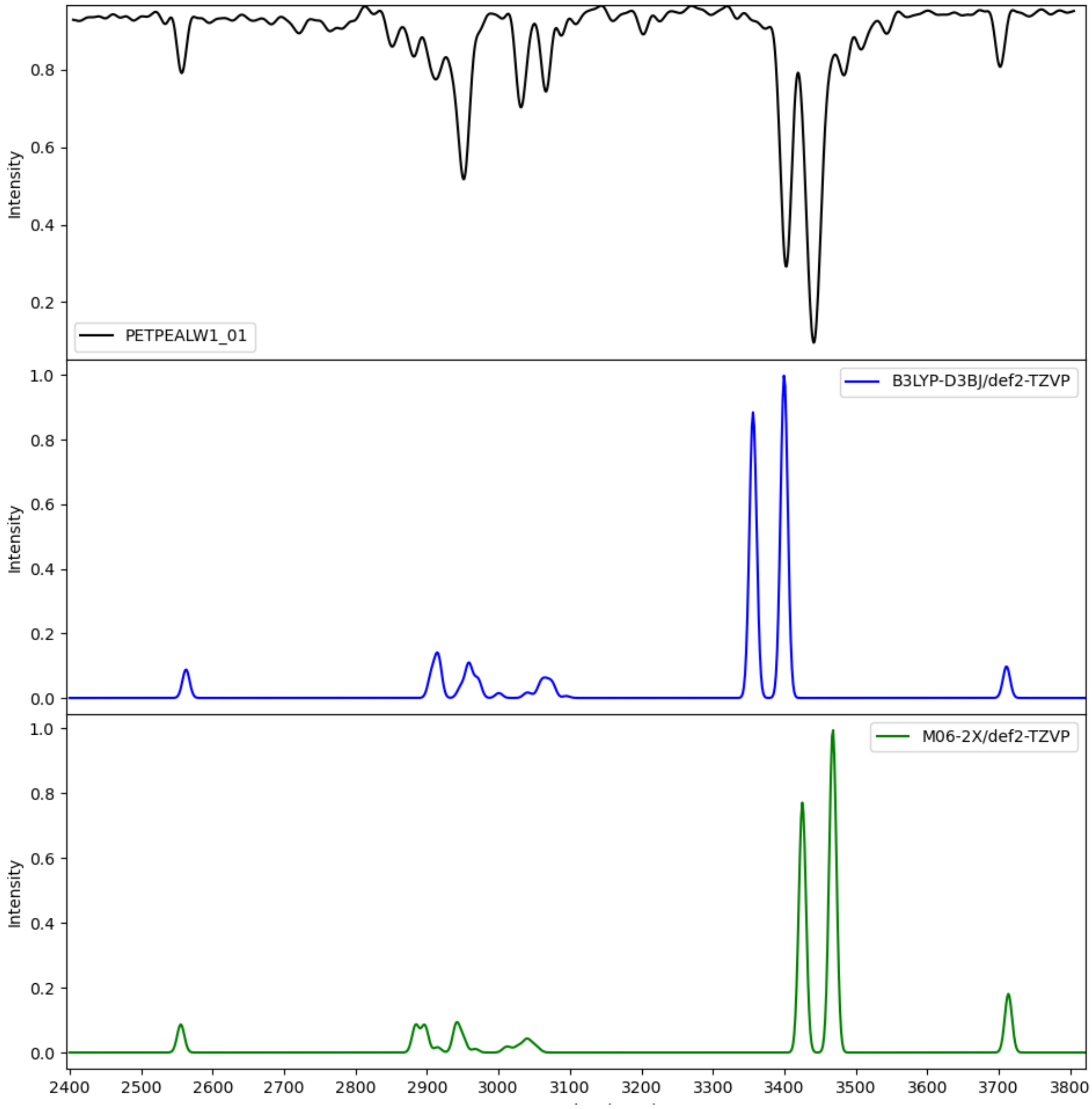

Figure S16. IDIR spectrum of the observed isomer of PEAL$_2$-W$_1$ compared to the simulated IR spectra at the B3LYP-D3BJ/def2-TZVP and M06-2X/def2-TZVP levels. A scaling factor of 0.963 was used for B3LYP-D3BJ/def2-TZVP and 0.951 for M06-2X/def2-TZVP.

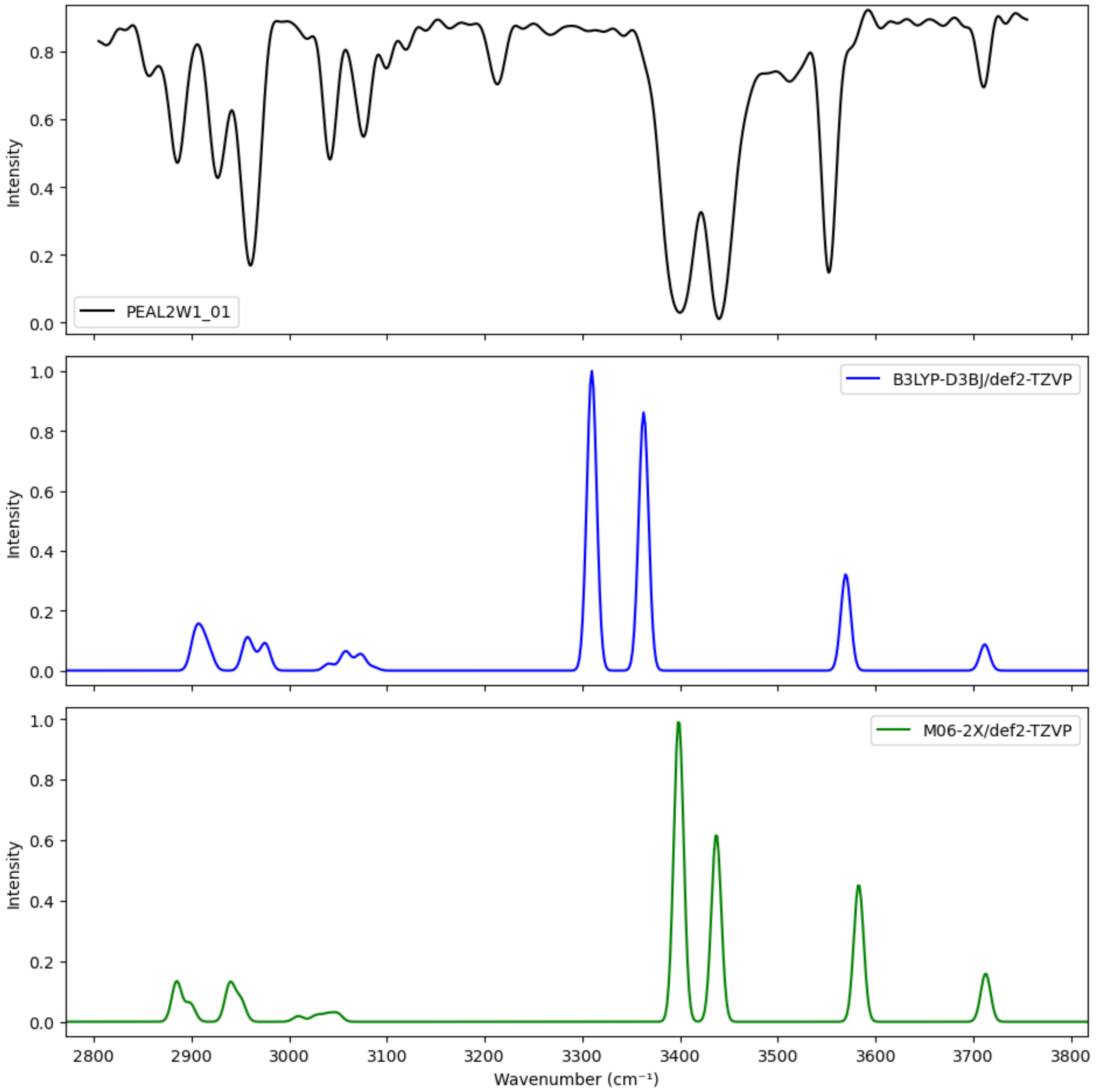

Figure S17. IDIR spectrum of the observed isomer of PEAL-PEA-W_1 compared to the simulated IR spectra at the B3LYP-D3BJ/def2-TZVP and M06-2X/def2-TZVP levels. A scaling factor of 0.963 was used for B3LYP-D3BJ/def2-TZVP and 0.951 for M06-2X/def2-TZVP.

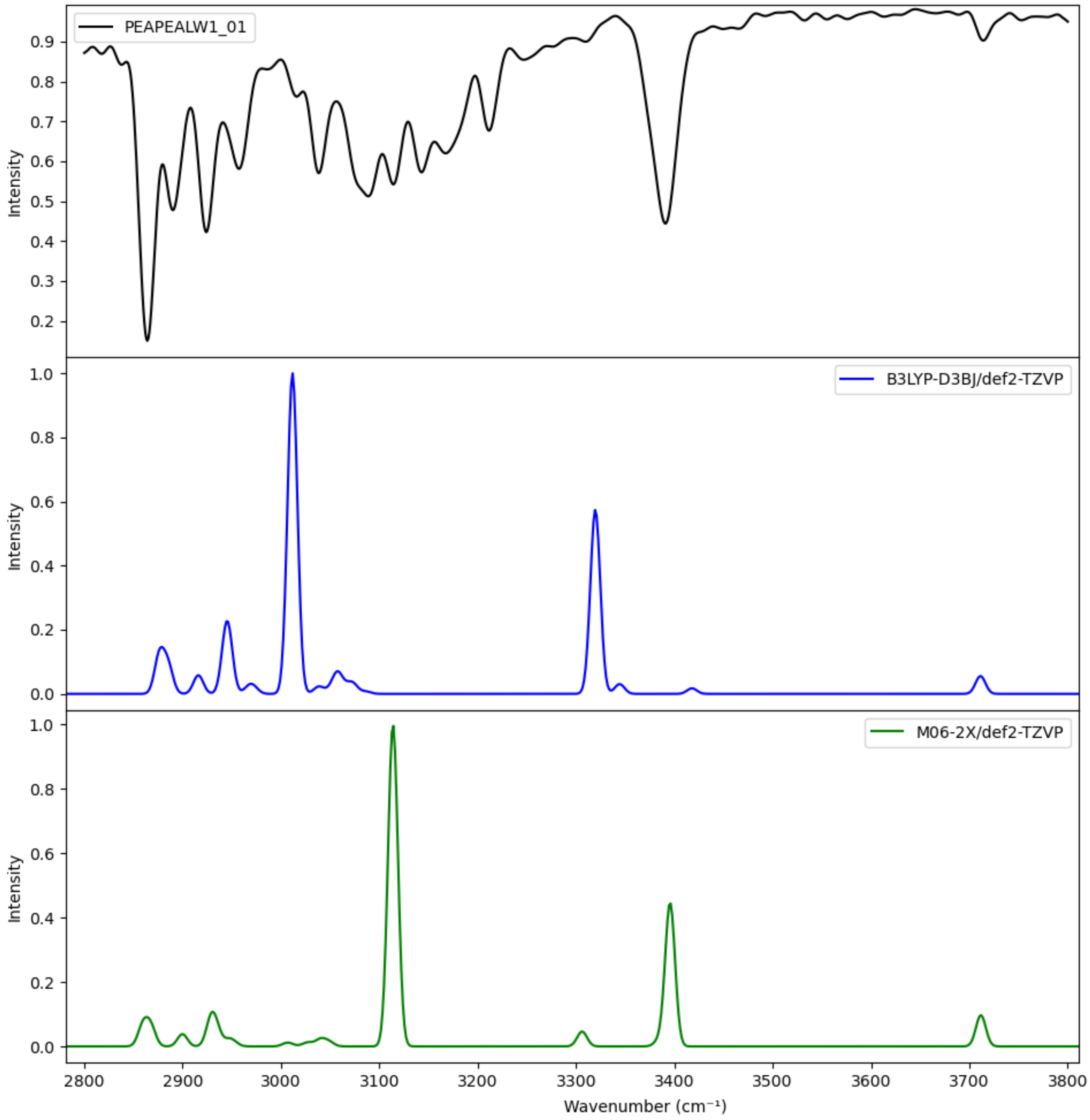

Figure S18. 2D NCI plots of the monohydrates compared with their corresponding dimers.

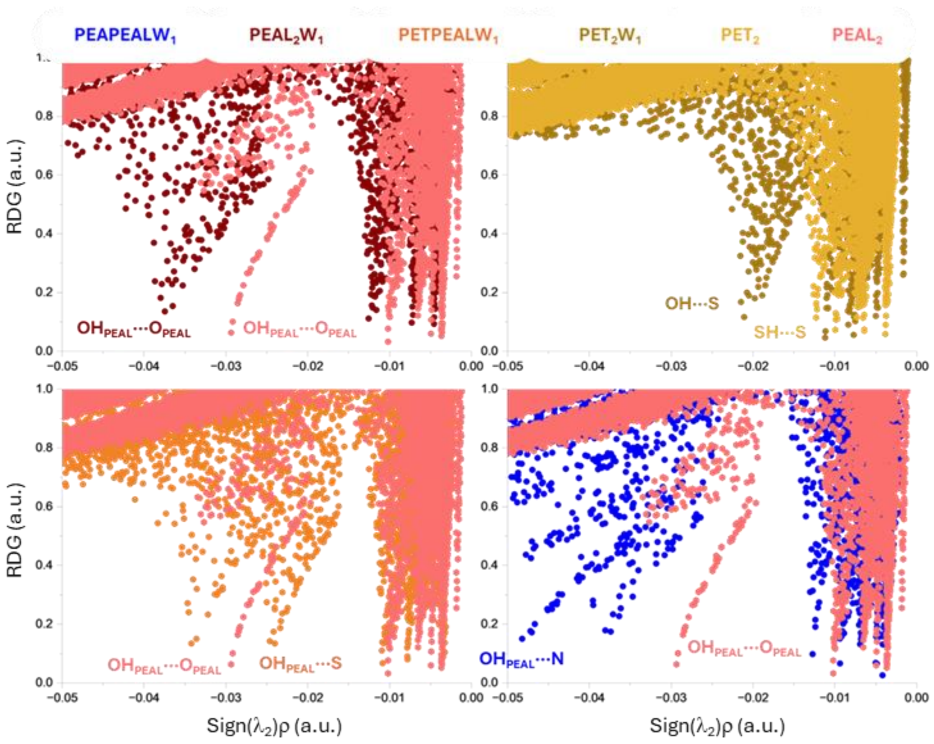

Figure S19. Enlarged version of **Figure 4** in the main text. a) Surface representation of the NCIs and b) 2D-NCIplot analysis of the monohydrated dimers: PET_2-W_1, PEAL-PET-W_1, $PEAL_2$-W_1, and PEAL-PEA-W_1. Data obtained from the calculations at B3LYP-D3BJ/def2-TZVP level.

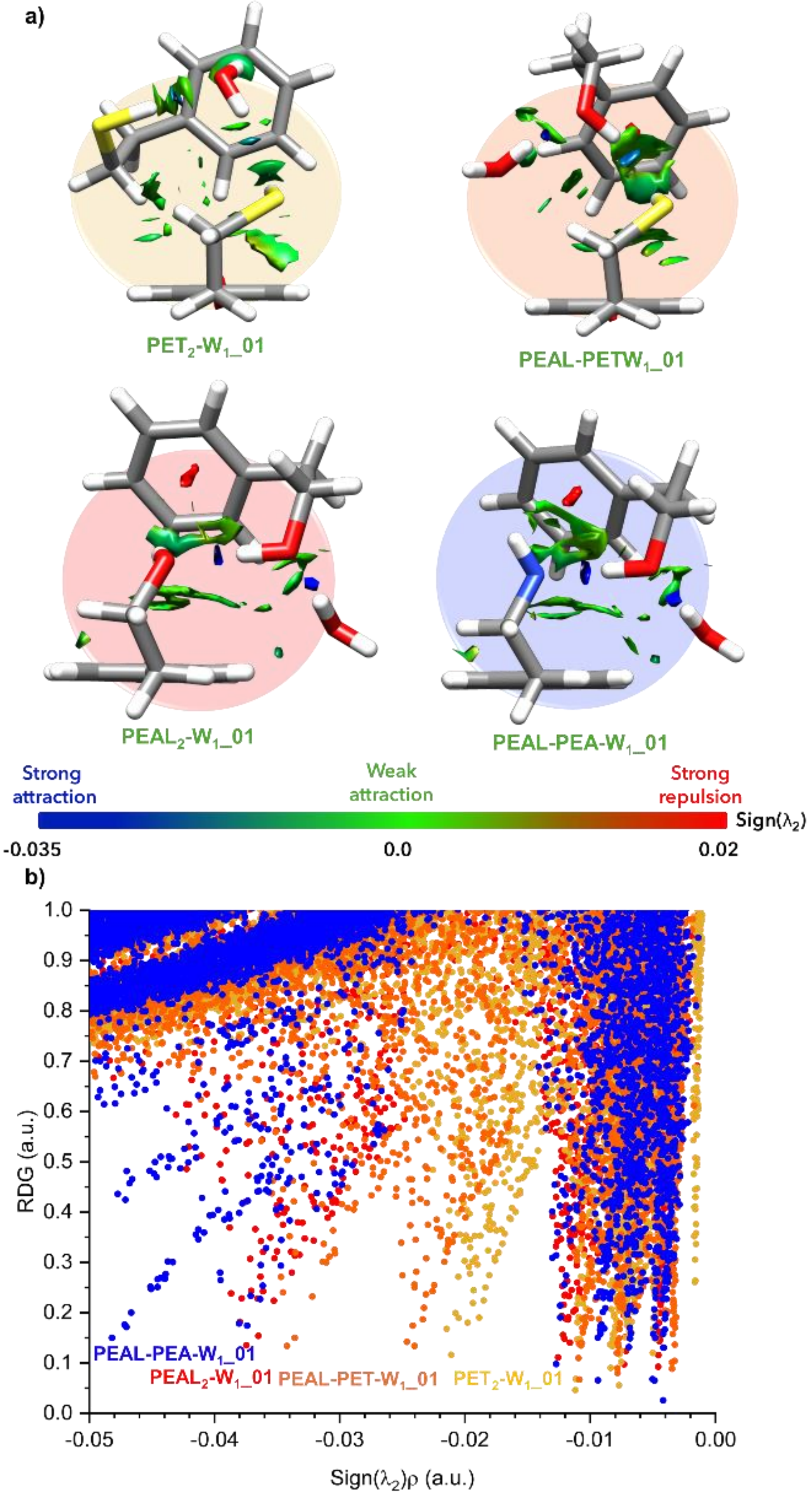

Figure S20. Strength of the hydrogen bonds in the PEAL-based monohydrates vs. the position of their OH stretching. The hydrogen bond strength was calculated using the bond critical points at B3LYP-D3BJ/def2-TZVP level. PEAL's OH stretching was considered as reference value.

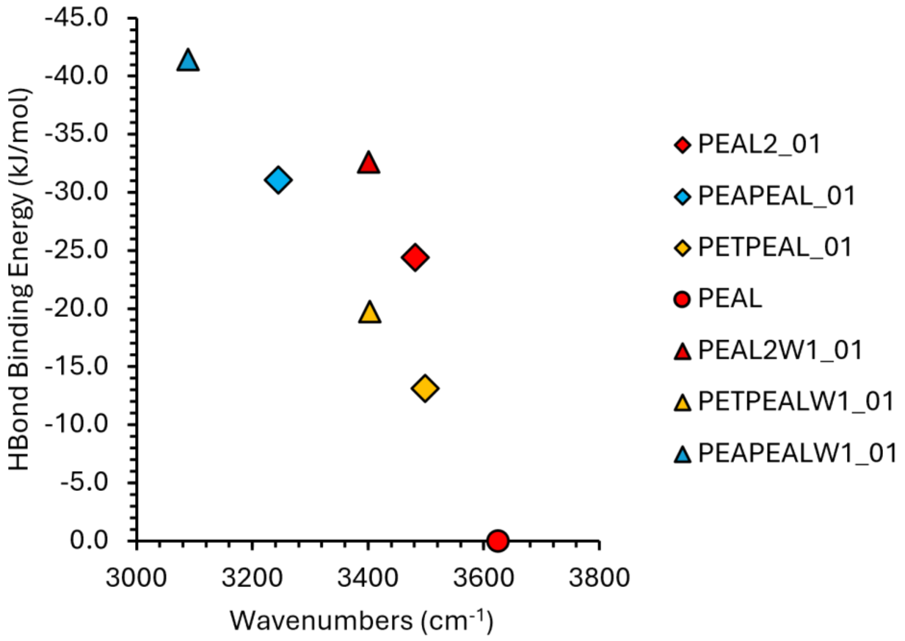

Table S1. Electron density at the bond critical point (ρ(rBCP), in atomic units) and the estimated binding energy (BE, in kJ·mol^{-1}) for the hydrogen bonds present in the assigned monohydrates. Three types of interactions are considered: $O_wH\cdots X$ (water hydrogen to heteroatom), XH···X (heteroatom hydrogen to heteroatom), and XH···π (heteroatom hydrogen to aromatic ring). The first three columns report ρ(rBCP) values for each interaction type, while the next three columns provide the corresponding binding energies. These data were computed at the B3LYP-D3BJ/def2-TZVP level of theory.

.

	ρ(rBCP) $O_wH\cdots X$ (a.u.)	ρ(rBCP) XH···X (a.u.)	ρ(rBCP) XH···π (a.u.)	BE OwH···X (kJ/mol)	BE XH···X (kJ/mol)	BE XH···π (kJ/mol)
$PEAL_2$-W_1_01	$3.73\cdot10^{-2}$	$3.83\cdot10^{-2}$	$1.22\cdot10^{-2}$	-31.7	-32.6	-8.2
PET_2-W_1_01	$1.96\cdot10^{-2}$	$2.14\cdot10^{-2}$	$8.19\cdot10^{-3}$	-15.2	-16.9	-4.5
PEAL-PET-W_1_01	$3.44\cdot10^{-2}$	$2.45\cdot10^{-2}$	$1.10\cdot10^{-2}$	-29.0	-19.7	-7.1
PEAL-PEA-W_1_01	$3.89\cdot10^{-2}$	$4.77\cdot10^{-2}$	$9.42\cdot10^{-3}$	-33.2	-41.4	-5.7
$PEAL_2$		$2.95\cdot10^{-2}$	$1.02\cdot10^{-2}$		-24.4	-6.4
PET_2		$1.25\cdot10^{-2}$	$9.98\cdot10^{-3}$		-8.6	-6.2

Table S2. BE_{HB} together with the OH and SH stretching for the assigned monohydrates, computed at B3LYP-D3BJ/def2-TZVP level. Binding energy values in kJ/mol; vibrations in cm^{-1}.

	BE	OH Stretching	SH Stretching
$PEAL_2$-W_1_01	-32.6	3401	
PET_2-W_1_01	-16.9		2513
PEAL-PET-W_1_01	-19.7	3403	
PEAL-PEA-W_1_01	-41.4	3089	
$PEAL_2$	-24.4	3628	
PET_2	-8.6		2540

References

(1) León, I.; Lesarri, A.; Fernández, J. A. Evaluation of the Aggregation Process in a Mixture of Propofol and Benzocaine. *Physical Chemistry Chemical Physics* **2019**, *21* (7), 3537–3544.

(2) Leon, I.; Cocinero, E. J.; Millán, J.; Jaeqx, S.; Rijs, A. M.; Lesarri, A.; Castaño, F.; Fernández, J. A. Exploring Microsolvation of the Anesthetic Propofol. *Physical Chemistry Chemical Physics* **2012**, *14* (13), 4398–4409.

(3) Halgren, T. A. MMFF VII. Characterization of MMFF94, MMFF94s, and Other Widely Available Force Fields for Conformational Energies and for Intermolecular-Interaction Energies and Geometries. *J Comput Chem* **1999**, *20* (7), 730–748.

(4) Halgren, T. A. Merck Molecular Force Field. II. MMFF94 van Der Waals and Electrostatic Parameters for Intermolecular Interactions. *J Comput Chem* **1996**, *17* (5–6), 520–552.

(5) Becke, A. D. Density-functional Thermochemistry. III. The Role of Exact Exchange. *J Chem Phys* **1993**, *98* (7), 5648–5652.

(6) Becke, A. D.; Johnson, E. R. A Density-Functional Model of the Dispersion Interaction. *Journal of Chemical Physics* **2005**, *123* (15), 154101.

(7) Grimme, S.; Ehrlich, S.; Goerigk, L. Effect of the Damping Function in Dispersion Corrected Density Functional Theory. *J Comput Chem* **2011**, *32* (7), 1456–1465.

(8) Zhao, Y.; Truhlar, D. G. The M06 Suite of Density Functionals for Main Group Thermochemistry, Thermochemical Kinetics, Noncovalent Interactions, Excited States, and Transition Elements: Two New Functionals and Systematic Testing of Four M06-Class Functionals and 12 Other Functionals. *Theor Chem Acc* **2008**, *120* (1–3), 215–241.

(9) Weigend, F.; Ahlrichs, R. Balanced Basis Sets of Split Valence, Triple Zeta Valence and Quadruple Zeta Valence Quality for H to Rn: Design and Assessment of Accuracy. *Physical Chemistry Chemical Physics* **2005**, *7* (18), 3297–3305.

(10) Boys, S. F.; Bernardi, F. The Calculation of Small Molecular Interactions by the Differences of Separate Total Energies. Some Procedures with Reduced Errors. *Mol Phys* **1970**, *19* (4), 553–566.

(11) Frisch, M. J.; Trucks, G. W.; Schlegel, H. B.; Scuseria, G. E.; et al. Gaussian 16, Revision C.01. Wallingford 2016.

(12) Contreras-García, J.; Johnson, E. R.; Keinan, S.; Chaudret, R.; Piquemal, J. P.; Beratan, D. N.; Yang, W. NCIPLOT: A Program for Plotting Noncovalent Interaction Regions. *J Chem Theory Comput* **2011**, *7* (3), 625–632.

(13) Johnson, E. R.; Keinan, S.; Mori-Sánchez, P.; Contreras-García, J.; Cohen, A. J.; Yang, W. Revealing Noncovalent Interactions. *J Am Chem Soc* **2010**, *132* (18), 6498–6506.

(14) Emamian, S.; Lu, T.; Kruse, H.; Emamian, H. *J Comput Chem.* **2019**, *40*, 2868–2881
